# Controlling ultralong room temperature phosphorescence in organic compounds with sulfur oxidation state†

**DOI:** 10.1039/d0sc04715e

**Published:** 2020-11-02

**Authors:** Zhen Xu, Clàudia Climent, Christopher M. Brown, Duane Hean, Christopher J. Bardeen, David Casanova, Michael O. Wolf

**Affiliations:** Department of Chemistry, University of British Columbia 2036 Main Mall Vancouver BC V6T 1Z1 Canada mwolf@chem.ubc.ca; Departamento de Física Teórica de la Materia Condensada, Universidad Autónoma de Madrid E-28049 Madrid Spain; Department of Chemistry, University of California Riverside 501 Big Springs Road Riverside California 92521 USA christob@ucr.edu; Donostia International Physics Center (DIPC) Paseo Manuel de Lardizabal 4 20018 Donostia Euskadi Spain david.casanova@ehu.eus; IKERBASQUE, Basque Foundation for Science 48013 Bilbao Euskadi Spain

## Abstract

Sulfur oxidation state is used to tune organic room temperature phosphorescence (RTP) of symmetric sulfur-bridged carbazole dimers. The sulfide-bridged compound exhibits a factor of 3 enhancement of the phosphorescence efficiency, compared to the sulfoxide and sulfone-bridged analogs, despite sulfone bridges being commonly used in RTP materials. In order to investigate the origin of this enhancement, temperature dependent spectroscopy measurements and theoretical calculations are used. The RTP lifetimes are similar due to similar crystal packing modes. Computational studies reveal that the lone pairs on the sulfur atom have a profound impact on enhancing intersystem crossing rate through orbital mixing and screening, which we hypothesize is the dominant factor responsible for increasing the phosphorescence efficiency. The ability to tune the electronic state without altering crystal packing modes allows the isolation of these effects. This work provides a new perspective on the design principles of organic phosphorescent materials, going beyond the rules established for conjugated ketone/sulfone-based organic molecules.

## Abstract 2

Sulfur lone pairs in bridged dimers enhance intersystem crossing and phosphorescence through orbital mixing and electrostatic screening.

## Graphical abstract

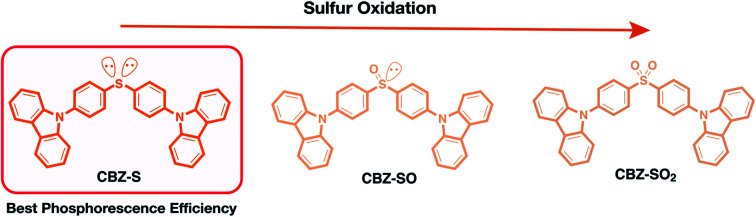

## Introduction

Phosphorescent materials are conventionally based on relatively rare and expensive inorganic elements (such as Ir, Pt and Eu),^1^ which limits their application in light-emitting devices.^2^ Recently, organic materials exhibiting room-temperature phosphorescence (RTP) have attracted significant attention.^3–5^ RTP provides the ability to utilize long-lived triplet states, yielding potential applications in optoelectronics such as organic light emitting displays (OLEDs),^6^ data encryption,^7,8^ and in chemical and biological sensing and imaging.^9,10^ Organic RTP emitters also exhibit advantages such as low cost, synthetic versatility, and stability.

**Fig. 1:**
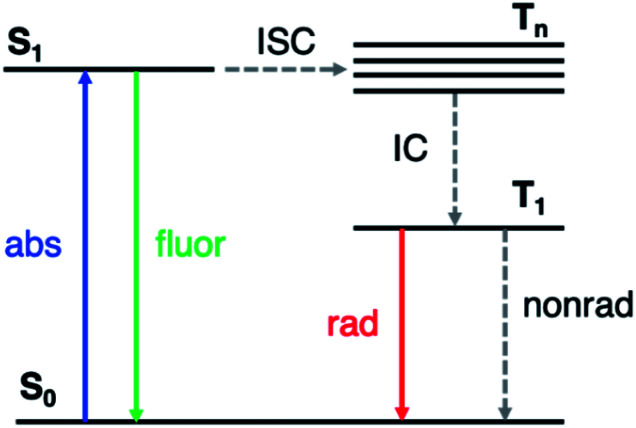
Simplified Jablonski diagram representing the relevant photophysics. ISC: intersystem crossing; IC: internal conversion.

Long-lived RTP in organic materials requires both high intersystem crossing (ISC) rates (from the first excited singlet state S_1_ to excited triplet states T_*n*_) and suppression of nonradiative decay from T_1_ to the ground state (S_0_) (Fig. 1). El Sayed's rule^11^ states that ISC is most efficient when the transition involves a change in molecular orbital type, *i.e.*, from ^1^(n, π*) to ^3^(π, π*) or from ^1^(π, π*) to ^3^(n, π*). In many organic molecules both S_1_ and T_*n*_ states have predominantly (π, π*) character, so one strategy to enhance RTP is to incorporate functional groups that induce (n, π*) character in one of the excited states. For example, Shuai and co-workers have shown computationally that ISC from S_1_ to T_*n*_ can be enhanced by increasing the (n, π*) transition component of the excited triplet state (T_*n*_) that is closest in energy to S_1_.^12^ Carbonyl and sulfone groups have been extensively employed to increase the (n, π*) character of the singlet and triplet states owing to the presence of lone pairs on the oxygen atoms.^13–22^ Once the T_1_ state is populated, its lifetime is determined by both radiative and nonradiative processes with the latter typically dominating in organic materials. The overall phosphorescence lifetimes are determined by the (π, π*) character of T_1_ and the nearby singlet states. Nonradiative decay to the ground state depends on spin orbit coupling (SOC) to S_0_, while the radiative rate depends on SOC to excited singlet states that can lend oscillator strength to T_1_. Thus, a greater degree of (π, π*) contribution in T_1_ reduces ISC from T_1_ to S_0_, and therefore increases the phosphorescence lifetime. Despite these insights into the excited state nature of RTP emitters, challenges remain on how to understand and control the lone pair involvement in the (n, π*) transitions and low-lying excited states, in order to design efficient and long-lived RTP materials.

We have previously reported a method for electronic state tuning in which sulfur groups with variable oxidation states control electronic properties in a symmetrical bichromophore system.^23^ In terthiophene dimers, a systematic increase in fluorescence quantum yields was observed upon oxidizing the sulfur bridge from sulfide (S), to sulfoxide (SO), and to sulfone (SO_2_). In solution, an intermediate charge transfer (CT) excited singlet state 
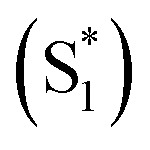
 was found with symmetry breaking character and variable degrees of charge localization. This CT 
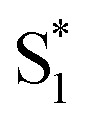
 state does not efficiently couple with the triplet states, resulting in a decrease in ISC efficiency. The sulfur lone pairs modulate the electronic coupling between the chromophores by electrostatic screening and lessen the amount of CT 
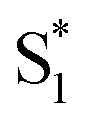
 character in the relaxed excited state.^24^ Upon oxidizing the sulfur atom, lone pair screening is reduced and the CT state is stabilized, preventing ISC and leading to enhanced fluorescence quantum yields. We have also demonstrated that the oxidation state at the sulfur bridge of conjugated homodimers, such as naphthalene or anthracene, controls their photochemistry by promoting photodimerization or enhancing the nonradiative decay through a conical intersection.^25,26^ The concept of tuning the electronics of a species by varying the sulfur oxidation state has since been applied to various molecular systems including oligomers, polymers and metal complexes.^27–32^

In the bridged terthiophene compounds, the bridging sulfur lone pairs (n(S)) are low in energy compared to the π HOMO of terthiophene, and thus do not participate significantly in the low-lying excited states. Their involvement is indirect, through the screening effect of the lone pairs. However, for higher bandgap chromophores like naphthalene and anthracene, the n(S) orbitals are able to mix with the chromophore HOMOs.^25,26^ Given the involvement of (n, π*) excited states in RTP, it would be intriguing to vary the n(S) component in sulfur-bridged chromophores using oxidation state, to control phosphorescence. There are two questions that are interesting to consider: (1) can the n(S) lone pairs influence intersystem crossing in phosphorescent organic compounds directly by increasing the singlet and/or triplet (n, π*) character or indirectly *via* the lone pair screening effect and creation of CT states?, and (2) since sulfone groups have been frequently used to enhance (n, π*) character in RTP compounds,^13,14,16,19–21^ how do the oxygen lone pair orbitals (n(O)) in the SO_2_ group affect the (n, π*) transition compared to the n(S) orbitals in sulfide?

Intermolecular interactions play a vital role in determining photophysical behavior and RTP efficiency in the solid state.^33,34^ The effects of intermolecular interactions on RTP have been intensively investigated, showing that by utilizing structural isomerism,^22,35^ polymorphism,^36^ and methylation,^20,21^ crystal packing modes can be altered, thereby tuning phosphorescence efficiencies and lifetimes. It is, however, notoriously difficult to predict crystal packing due to the possibility of polymorphs, even when using specific crystallization conditions, making it challenging to intentionally control intermolecular interactions. An alternate approach is to make electronic modifications to the individual chromophores, however such studies are rare,^37^ principally since it is difficult to modify chromophores without simultaneously altering crystal packing.

**Fig. 2:**
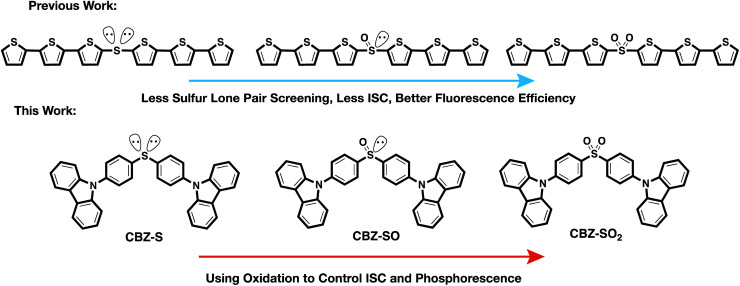
Design principle and chemical structures of sulfur-bridged dimers for fluorescent and phosphorescent materials.

To examine the effects of sulfur oxidation state on n(S) and n(O) involvement in RTP, we constructed carbazole-functionalized analogs of the sulfur-bridged terthiophene series (Fig. 2). Carbazole derivatives have been widely explored as triplet chromophores for both TADF and RTP.^3,13^ A key structural feature in this series of chromophores is the tetrahedral geometry of the sulfur center that enables near-identical packing modes, regardless of sulfur oxidation state. With specific functional groups, the (n, π*) and (π, π*) character can be tuned. We find that the lone pair orbital mixing and screening can have a substantial effect on the overall RTP efficiency, providing a new approach for optimizing these materials.

## Results and discussion

Sulfide CBZ-S was synthesized *via* electrophilic substitution of bis(4-bromophenyl)sulfane with 9*H*-carbazole (Scheme S1†). Oxidation of the sulfur was carried out by adding one equivalent of *meta*-chloroperoxybenzoic acid (*m*-CPBA) to a stirred CH_2_Cl_2_ solution of CBZ-S, yielding the sulfoxide CBZ-SO. Sulfone CBZ-SO_2_ was similarly synthesized through the addition of 2.2 equivalents of *m*-CPBA to CBZ-S. All compounds were characterized by ^1^H and ^13^C NMR spectroscopy and mass spectrometry. CBZ-S and CBZ-SO_2_ have been previously reported.^38,39^

**Fig. 3:**
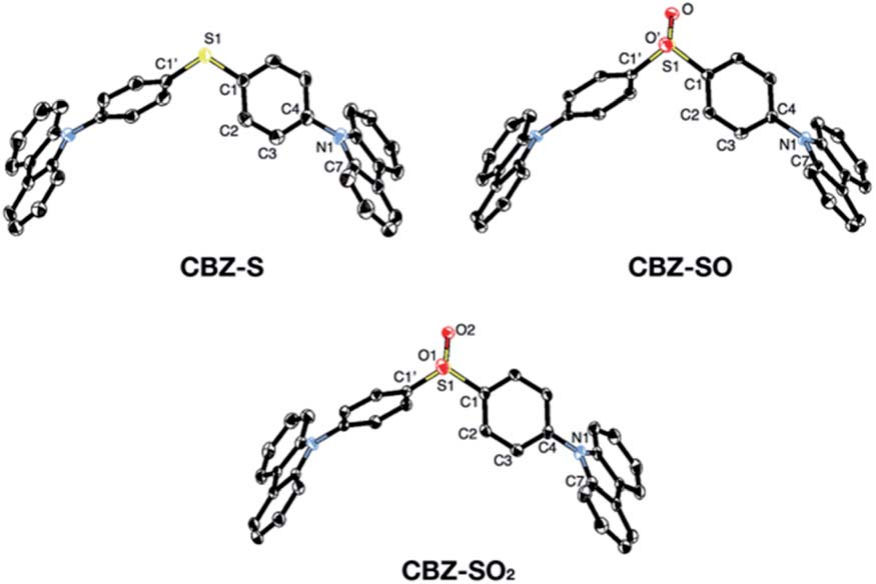
ORTEP representations of single-crystal structures of CBZ-S, CBZ-SO and CBZ-SO_2_. Ellipsoids are plotted at the 50% probability level. Hydrogen atoms are removed for clarity.

Solid-state structures of CBZ-S, CBZ-SO and CBZ-SO_2_ were obtained by single-crystal X-ray diffraction (Fig. 3). All species crystallize without solvent inclusion in the *C*2/*c* space group and have similar unit cell parameters. A previous structure of CBZ-SO_2_ contains lattice MeOH with different unit cell parameters and packing reported.^38^ The sulfur centers of CBZ-S, CBZ-SO and CBZ-SO_2_ all adopt pseudo-tetrahedral geometries with C1–S–C1′ bond angles of 104.8°, 100.3° and 106.2°, respectively. CBZ-SO and CBZ-SO_2_ display C1–S–O angles of 103.9° and 107.8°, respectively. CBZ-SO shows two oxygen sites in the crystal structure with 50% occupancy in each site, confirming the sulfoxide species. The phenyl and carbazole rings are twisted relative to each other in all three species, with torsion angles of 118.5°, 125.4° and 123.6° in CBZ-S, CBZ-SO and CBZ-SO_2_, respectively.

The crystallographic data show that with oxidation of the sulfur atom, the molecular geometries of these compounds remain largely unchanged. Molecular structures optimized by means of density functional theory (DFT) are in good agreement with the molecular geometries in the crystal (Table S2†). The main discrepancy corresponds to the relative orientation between the two phenyl rings in CBZ-SO and CBZ-SO_2_. Optimization of the free molecule favors the formation of two and four SO⋯H hydrogen bonds, respectively (Fig. S11†), with atomic separations smaller than the sum of the van der Waals radii.^40^ Such interaction results in small phenyl–phenyl dihedral angles. In the crystal, packing forces appear to prefer the symmetric disposition of the two carbazole fragments, *i.e.*, small dihedral angles (Table S2†), at the expense of hydrogen bond formation (one and two for CBZ-SO and CBZ-SO_2_ respectively).

**Fig. 4:**
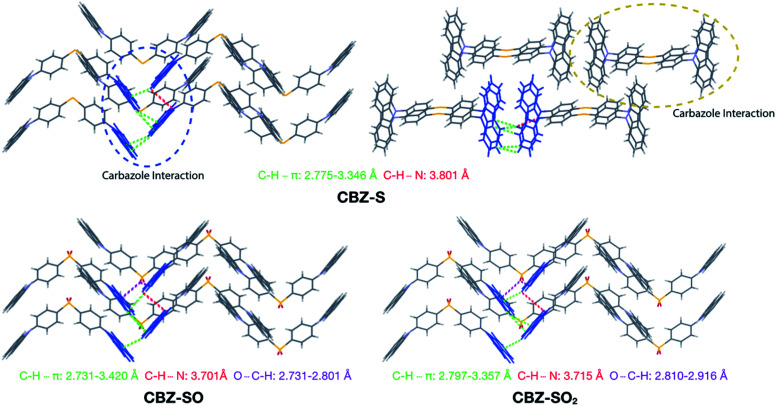
Packing modes and intermolecular interactions of CBZ-S, CBZ-SO and CBZ-SO_2_.

Intermolecular interactions can affect the solid-state photoluminescence properties of the species. As such, crystallographic packing diagrams have been carefully examined (Fig. 4). All three compounds (CBZ-S, CBZ-SO and CBZ-SO_2_) adopt similar packing modes in which the molecules stack on top of each other. There are also numerous interactions between adjacent carbazole groups. The CH⋯π distances between adjacent carbazole groups are similar in all three compounds: 2.775 to 3.346 Å in CBZ-S 2.731 to 3.420 Å in CBZ-SO and 2.797 to 3.357 Å in CBZ-SO_2_. These findings confirm that molecular packing does not change drastically between the three structures, despite different sulfur oxidation states. This allows the electronic effects between compounds of different sulfur oxidation states to be assessed independently of the packing modes.

The absorption and photoluminescence spectra of sulfide (CBZ-S), sulfoxide (CBZ-SO) and sulfone (CBZ-SO_2_) in cyclohexane (CH_*x*_), CH_2_Cl_2_ and MeCN solutions are shown in Fig. S1.† Structured features between 275 nm and 350 nm are present in all compounds. Electronic structure calculations characterize the lowest optical transitions in CBZ-S and CBZ-SO mainly as linear combinations of local (π, π*) excitations on the two carbazole moieties (Fig. S11†).^41^ Excitation energies to S_1_ and S_2_ are nearly identical to the lowest singlet–singlet transition in 9-phenylcarbazole (Tables S3 and S4†), suggesting weak intermonomer exciton coupling. Interestingly, the lowest dipole allowed excitation in CBZ-SO_2_ exhibits significant carbazole-to-phenyl electron transfer character and is red-shifted with respect to CBZ-S and CBZ-SO. The relative intensities of the structured peaks are only altered slightly with oxidation at the bridging sulfur, or with solvent polarity.

Oxidizing the sulfur atom also causes a red-shift of the emission profiles from CBZ-S, CBZ-SO to CBZ-SO_2_, in agreement with computed vertical de-excitation energies (Table S5†). With increasing solvent polarity, a <10 nm red shift is seen in CBZ-S from CH_*x*_ to MeCN and the emission bands became less structured. In contrast, the sulfoxide and sulfone compounds, CBZ-SO and CBZ-SO_2_, both show a strong solvent dependence. They display broad, featureless photoluminescence spectra in CH_2_Cl_2_ and MeCN solutions, significantly red-shifted compared to spectra in CH_*x*_ solutions. CBZ-SO has an emission maximum at 358 nm in CH_*x*_ solution, and 416 nm in MeCN, while an emission maximum at 369 nm in CH_*x*_ and 440 nm in MeCN was observed for CBZ-SO_2_. This is indicative of CT character in both compounds. The excited state CT character is enhanced by an increase in sulfur oxidation level. Meanwhile, the photoluminescence quantum yields (PLQYs) increase from 0.12 in CBZ-S, to 0.38 in CBZ-SO, and 0.64 in CBZ-SO_2_. These two findings indicate that the increase in the CT character through bridge oxidation can reduce the competition from other non-radiative decay pathways such as ISC.^24^

Computational simulation of the structural relaxation on the excited state potential energy surface (PES) indicates the presence of excited state minima with sizeable CT character between two linked monomers in SO and SO_2_-bridged dimers. The optimized S_1_ state in CBZ-S corresponds to localized or delocalized (π, π*) excitations with only small structural rearrangements with respect to the ground state geometry (Fig. S13†). In contrast, the excited state PESs of CBZ-SO and CBZ-SO_2_ present strong through-space orbital overlap between the linked monomers, which resembles the electronic structure of excimers and therefore can be described as excimer-like states (Fig. S14 and S15†).^25,26^ The increase in interchromophoric interaction and CT character of excited states with the oxidation state of the linker is consistent with our previous findings^23^ and computational studies showing a similar decrease of the electronic screening effect of sulfur lone pairs from S, to SO and SO_2_-bridged dimers.^24^

Before carrying out solid-state photophysical measurements, powder X-ray diffraction (pXRD) patterns of CBZ-S and CBZ-SO were collected in order to determine the crystallinity and phase of the bulk samples (Fig. S3 and S4†). The experimental patterns match very well with the simulated patterns generated from the single-crystal structures, confirming that bulk powder samples of these compounds have the same packing modes as the single crystals. Due to polymorphism in CBZ-SO_2_, the bulk CBZ-SO_2_ powder exhibits a more complicated pXRD pattern than the simulated patterns calculated from the crystal structure, and attempts to obtain phase-pure powders were unsuccessful in this case. Thus, for solid-state photophysical measurements of CBZ-SO_2_, single-crystal samples were used.

**Fig. 5:**
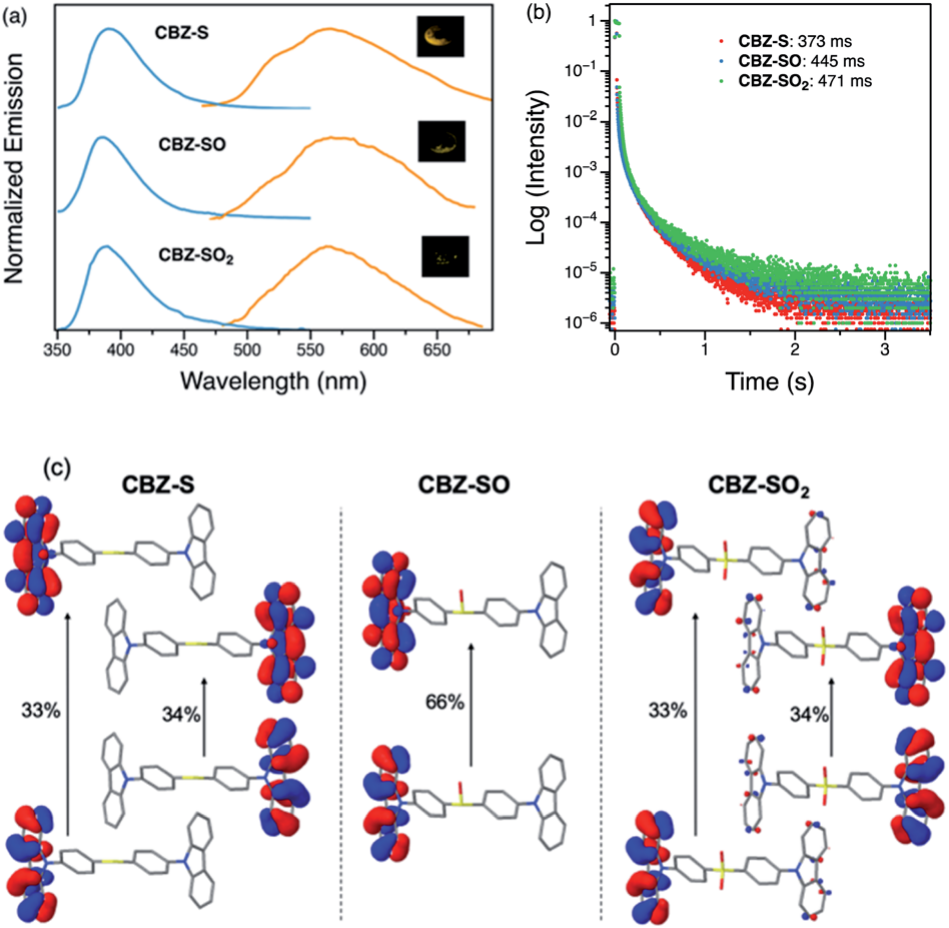
(a) Steady-state (blue) and time-resolved photoluminescence spectra with 1 ms delay (orange) of CBZ-S, CBZ-SO and CBZ-SO_2_ crystalline samples under ambient conditions at 298 K. Inset: photographs of CBZ-S, CBZ-SO and CBZ-SO_2_ crystalline samples after UV excitation is removed. (b) Phosphorescence decay profiles of CBZ-S, CBZ-SO and CBZ-SO_2_ crystalline samples under ambient conditions and the lifetimes of the longest components. (c) Main natural transition orbitals of the T_1_ state for the solid-state (crystal) molecular structure of CBZ-SO*_n_* obtained at the ωB97X-D/6-31+G(d) level. Hole/electron weight of the contributions indicated in %.

Photophysical properties of CBZ-S, CBZ-SO and CBZ-SO_2_ in the solid state were evaluated by photoluminescence spectroscopy (Fig. 5a). The photophysical data are summarized in Table S1.† All three compounds emit with very similar peak fluorescence maxima in the steady state, possibly due to the similar packing modes. CBZ-S shows a broad fluorescence band with a peak at 390 nm while CBZ-SO and CBZ-SO_2_ both have emission maxima at 388 nm. Furthermore, CBZ-SO_2_ exhibits a ∼30 nm blue shift compared to emission in solution and to the other previously reported polymorph in the solid state.^38^ This can be attributed to the molecular rigidity imposed by the crystal environment with respect to the molecular relaxation in solution (Fig. S17†). Excited state calculations of molecular dimers in the crystal indicate weak intermolecular interactions for the low-lying singlet and triplet states (Table S7†), suggesting that molecular structure has a larger impact on the photophysical properties of CBZ-SO*_n_* than crystal packing.^42^ The PLQY data in the solid state shows a similar trend to the solution data. CBZ-SO_2_ has the highest fluorescence efficiency in the solid state (0.75). However, CBZ-SO (0.21) is slightly less emissive than CBZ-S (0.27).

When a 1 ms delay is employed to collect the long-lived emission spectra, all three species exhibit phosphorescence bands at ∼565 nm, well-separated from the fluorescence signals. Computations of CBZ-SO*_n_* in the crystal molecular structure characterize the lowest triplet as a (π, π*) state on the carbazole moieties (Fig. 5c), with a very similar vertical energy to the ground state for all three molecules (Tables S5 and S6†) and for 9-phenylcarbazole (Table S3†). Optimization on the triplet potential energy surface results in the localization of the two unpaired electrons on one of the carbazole fragments (Fig. S16†). With increasing sulfur oxidation state, the energy levels of the first excited singlet and triplet states do not change significantly in the solid state. Impressively, after removal of the UV excitation source, a yellow afterglow was observed with the naked eye for all three compounds, indicative of ultralong persistent phosphorescence.

The solid-state phosphorescence properties were further investigated using photoluminescence lifetime measurements at room temperature. CBZ-S, CBZ-SO and CBZ-SO_2_ all show multi-exponential lifetimes with the longest components at 373 ms, 445 ms and 471 ms, respectively (Fig. 5b). CBZ-SO and CBZ-SO_2_ exhibit marginally longer lifetimes than CBZ-S. The additional intermolecular interactions between the oxygen atom and carbazole units might help rigidify the molecular matrix, thereby prolonging the phosphorescence lifetimes. However, such small changes could also be the result of crystal quality. Regardless, the introduction of oxygen atoms by sulfur oxidation has only a minimal effect on the phosphorescence lifetime.

Low-temperature studies in the solid state were also carried out to gain insight into the photophysical behavior of CBZ-S, CBZ-SO and CBZ-SO_2_. A new peak at 480 nm emerges in the steady-state photoluminescence spectra of CBZ-S at 77 K, while CBZ-SO and CBZ-SO_2_ only display the higher-energy (∼390 nm) luminescence peak already present at room temperature. The 1 ms delayed photoluminescence spectra of all three species show phosphorescence between 450–650 nm, shifted to higher energy compared to the room temperature phosphorescence; this can be attributed to the more compact packing.^21^ The match between the spectrum of CBZ-S obtained with a 1 ms time delay and the low energy peak in the steady-state spectrum verifies that this feature is phosphorescence. Fluorescence and phosphorescence are therefore both observed in CBZ-S with approximately comparable intensities at 77 K. Measured lifetimes of the three species range from 1660 to 2460 ms, demonstrating that at low temperature, molecular motions have been further suppressed and the lifetimes prolonged. CBZ-S, CBZ-SO and CBZ-SO_2_ all display an ultralong green afterglow, lasting more than five seconds by eye. The photophysical properties of CBZ-S match those in a recently published report.^39^

Measuring PLQYs using a xenon lamp source and an integrating sphere can sometimes give inaccurate values when emission intensity is extremely low, such as the RTP in this case. To enable determination of relative phosphorescence efficiencies for the three compounds, a 320 nm Nd:YAG laser was used as the excitation source and triplet photons emitted in the time range from 60 μs to 110 ms were collected. In order to minimize the influence of solid sample compactness and sample positioning between samples of three different compounds, the same laser was also used to measure each sample without any time delay immediately after the time-resolved measurements, allowing integration of the fluorescence from the sample. A scaling factor for phosphorescence intensity was calculated using the solid-state PLQYs of CBZ-S, CBZ-SO and CBZ-SO_2_, divided by their corresponding fluorescence intensity integration. As shown in Fig. 7, the scaled phosphorescence enables the relative phosphorescence efficiency of the three compounds to be evaluated. The calibrated integration ratio is 3 : 0.5 : 1 for CBZ-S to CBZ-SO to CBZ-SO_2_.

**Fig. 6:**
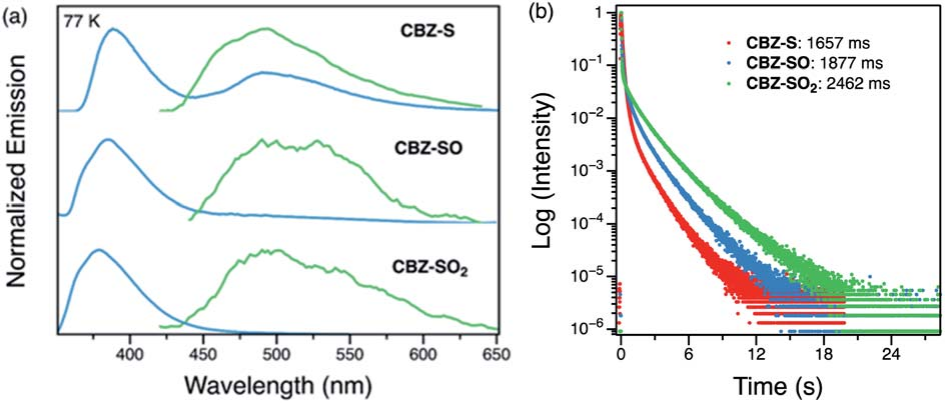
(a) Steady-state (blue) and time-resolved photoluminescence spectra with 1 ms delay (green) of CBZ-S, CBZ-SO and CBZ-SO_2_ crystalline samples at 77 K. (b) Phosphorescence decay profiles of CBZ-S, CBZ-SO and CBZ-SO_2_ crystalline samples at 77 K, with the lifetimes of the longest components stated.

**Fig. 7:**
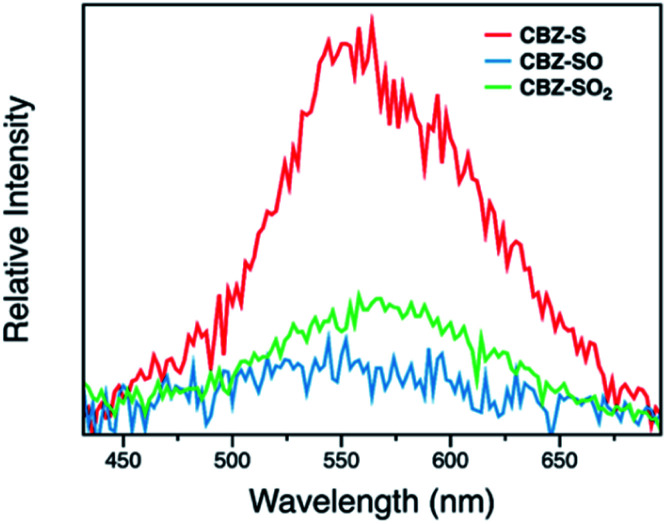
Time-resolved photoluminescence spectra (60 μs to 110 ms time range) of CBZ-S, CBZ-SO and CBZ-SO_2_ crystalline samples under ambient conditions at 298 K. The intensity is corrected by fluorescence integration under the same excitation condition as time-resolved measurements and PLQYs.

The surprising aspect of the data in Fig. 5–7 is that even though CBZ-S has the shortest lifetime, it has the largest phosphorescence QY (*ϕ*_ph_). To gain insight into this observation, we consider the kinetic origins of the QY. The observed *ϕ*_ph_ depends on both intersystem crossing between S_1_ and T_*n*_ and phosphorescence radiative decay rate:1
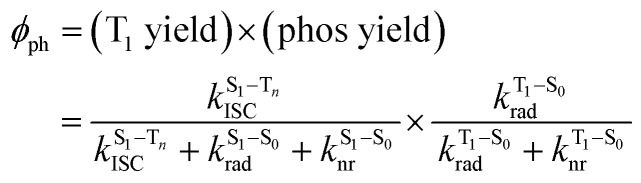
Here 
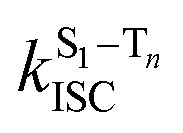
 is the intersystem crossing rate, 
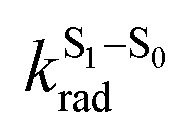
 is the radiative decay rate of S_1_, 
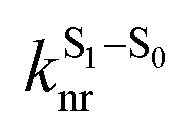
 is the nonradiative decay rate of S_1_, 
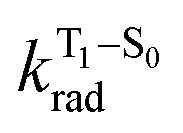
 is the radiative rate of the phosphorescence transition, and 
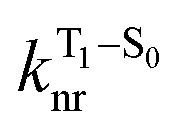
 is the nonradiative decay rate of T_1_. The intersystem crossing rate controls the population of the triplet manifold and is proportional to the square of the SOC between S_1_ and T_*n*_. Electronic structure calculations predict strong SOCs (on the order of few cm^−1^) between the lowest singlet and energetically close excited triplet states of CBZ-SO (Table S8 and Fig. S19†), similar to calculations by Varathan and co-workers.^43,44^ The strongest couplings are obtained for the triplets with significant contribution of the n(S) orbital, which is energetically stabilized through orbital mixing with the oxygen lone pairs (Fig. S20†). Such a synergic effect is not present in the S-bridged dimer, resulting in weaker singlet–triplet interactions. The weakest S_1_/T_*n*_ SOCs are computed in CBZ-SO_2_, which can be attributed to the lack of sulfur lone-pairs in the bridge. The calculated trend in ISC is consistent with the qualitative trend in experimental solid-state PLQY values, with CBZ-SO_2_ having the highest PLQY presumably due to the smallest 
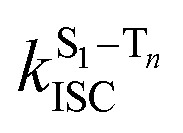
.

It is worth noting that our calculations have been done at the geometry found in the crystal structure, *i.e.*, they do not include possible dynamical effects after photoexcitation. Although we did not attempt to simulate geometrical relaxation in the solid state, computation of molecular excited state minima suggests the formation of S_1_ states with strong CT character in CBZ-SO and CBZ-SO_2_, but not in CBZ-S (Table S5 and Fig. S13–S15†). Therefore, we argue that the presence of sulfur lone pairs in S and SO linkers can potentially enhance ISC due to strong S_1_/T_*n*_ SOCs, while structural relaxation on the excited state PES of SO and SO_2_-bridged dimers might hinder intersystem crossing through an increase in interchromophoric interactions and the CT character of the singlet exciton.^24^ Although it is difficult to predict the relative importance of these two effects, we hypothesize that their combination might favor ISC in the S dimer relative to in the SO and SO_2_-bridged dimers. This rationalizes the enhanced phosphorescence from the sulfide bridged CBZ dimers, and the lower phosphorescence seen in the sulfoxide and sulfone analogs.

**Fig. 8:**
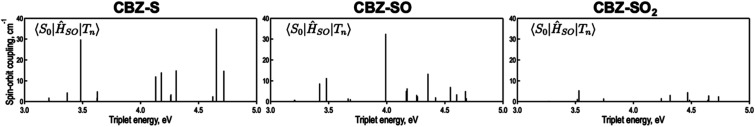
Spin orbit couplings (in cm^−1^) between excited triplet states and S_0_ of CBZ-SO*_n_* for the crystal molecular structure calculated at the ωB97X-D/6-31+G(d) level.

The second term in eqn (1) describes triplet state emission after T_*n*_ → T_1_ internal conversion. The phosphorescence yield depends on the radiative and nonradiative rates of the lowest triplet T_1_, with the latter being much faster and typically controlling the phosphorescence lifetime in organic compounds.^45^ The nonradiative decay rate 
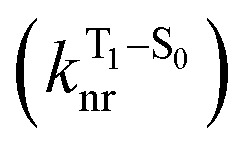
 will depend on packing, the presence of defects in the solid-state structures, and the electronic character of T_1_. The single-crystal structures of CBZ-S, CBZ-SO and CBZ-SO_2_ show that intermolecular packing in the three species is nearly identical, so sulfur oxidation state does not significantly affect how these molecules pack and interact with each other in the solid state. This suggests that variation of 
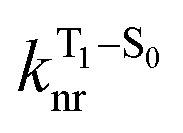
 will be directly related to changes in T_1_/S_0_ SOCs driven by (small) differences in the nature of the lowest triplet. Quantum chemical calculations for CBZ-SO*_n_* molecules show rather high SOCs between lower excited triplets and the ground state singlet (Fig. 8), in particular for those triplets with sizeable electronic contributions from the sulfur electron lone pairs. Decay within the triplet manifold drives the system to the T_1_ state with a markedly (π, π*) nature for the three dimers (Fig. 5c), and with small SOCs to the ground state singlet in all systems, which might allow for large phosphorescence lifetimes. The strength of S_0_/T_1_ couplings decreases with the oxidation of the bridge, that is 1.8 cm^−1^ (CBZ-S), 0.7 cm^−1^ (CBZ-SO) and 0.2 cm^−1^ (CBZ-SO_2_). These results suggest that oxidation of the bridge decreases the nonradiative rate back to the ground state 
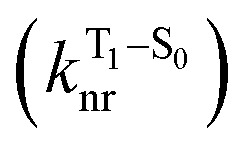
, resulting in an overall increase of the triplet lifetime, as observed experimentally. The strong (π, π*) character of T_1_ in all three systems results in small radiative rates 
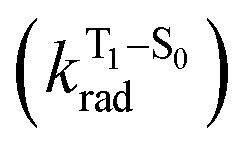
, with oscillator strengths for the emission from the lowest triplet states calculated in the order of 10^−8^ to 10^−9^ (Table S9†).

**Fig. 9:**
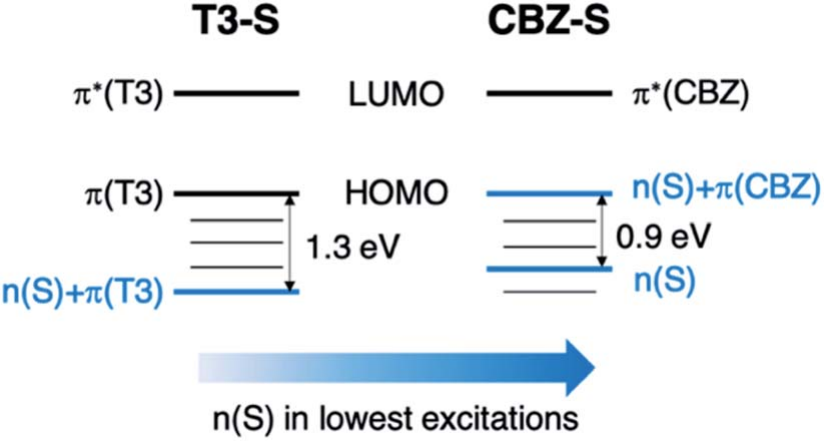
Schematic comparison of frontier orbitals in terthiophene (T3-S) and carbazole (CBZ-S) sulfide-bridged chromophores.

Compared to the terthiophene compounds, the n(S) lone pairs in the CBZ series have much stronger orbital mixing with the highest occupied π-orbitals owing to the closer energy levels (Fig. 9). The involvement of n(S) lone pairs in the low-lying excited states of CBZ-S and CBZ-SO potentially enhances T_1_–S_0_ and S_1_–T_*n*_ SOCs with respect to their terthiophene counterparts. It is worth highlighting that the n(O) orbitals in SO_2_ lie at much lower energies and do not contribute to SOC between low-lying triplet excited states and the ground state.

## Conclusions

Three sulfur-bridged carbazole compounds (CBZ-S, CBZ-SO and CBZ-SO_2_) are reported in which the photophysical properties can be tuned by varying the oxidation state at the sulfur center. The photophysical behavior of these compounds in solution parallels other sulfur-bridged organic chromophores, whereby fluorescence quantum yields are enhanced as the sulfur oxidation state is raised from sulfide to sulfoxide to sulfone. In the solid state, however, phosphorescence properties follow an opposing trend, in which the sulfide CBZ-S exhibits the highest phosphorescent efficiency in the series.

Sulfone groups are commonly used building blocks in organic luminescent materials, particularly in those that utilize triplet excitons (such as materials for RTP and TADF). This is consistent with El Sayed's rule which predicts that sulfone groups can enhance intersystem crossing by the presence of (n, π*) character in the singlet excited states. Here, however, we show that in SO_*n*_-bridged conjugated dimers sulfur electron lone-pairs are crucial to enhance S_1_–T_*n*_ SOCs. The synergic effect of sulfur and oxygen lone pairs induces strong couplings in CBZ-SO, while for the SO_2_ bridge the lone pairs of the two oxygen atoms are too low in energy to be involved in the photophysics. On the other hand, dynamical effects might contribute to hinder ISC to the triplet manifold in the sulfoxide and sulfone dimers. Molecular relaxation on the S_1_ PES of the SO and SO_2_-bridged dimers might drive the system to the formation of excimer-like states with strong CT character. The contribution of CT states is hindered in CBZ-S due to the electron screening effect. These two effects help to rationalize the phosphorescence enhancement in sulfide-containing species (CBZ-S), going beyond the rules established for the phosphorescence in conjugated ketone/sulfone-based organic molecules.^12^ These results support the conclusion that the increased ISC afforded by the sulfide bridge enhances the phosphorescence efficiency. Sulfone-bridged species have been extensively explored as TADF and RTP emitters,^13,14,16,19–21^ while sulfide-bridged compounds have been largely neglected for these applications.^10^ These findings provide a novel design strategy for triplet-utilizing luminescent materials. Previous RTP strategies have focused on tuning the molecular packing of the species, with electronic effects seldom considered. The ability to easily modify the electronic properties, without disturbing the intermolecular interactions or singlet and triplet energy levels, will be valuable in developing novel RTP emitters.

## Conflicts of interest

There are no conflicts of interest to declare.

## Supplementary Material

SC-012-D0SC04715E-s001

SC-012-D0SC04715E-s002

## References

[cit1] Evans R. C., Douglas P., Winscom C. J. (2006). Coord. Chem. Rev..

[cit2] Baldo M. A., Thompson M. E., Forrest S. R. (1999). Pure Appl. Chem..

[cit3] Forni A., Lucenti E., Botta C., Cariati E. (2018). J. Mater. Chem. C.

[cit4] Kenry, Chen C., Liu B. (2019). Nat. Commun..

[cit5] Li Q., Tang Y., Hu W., Li Z. (2018). Small.

[cit6] Cai X., Qiao Z., Li M., Wu X., He Y., Jiang X., Cao Y., Su S.-J. (2019). Angew. Chem., Int. Ed..

[cit7] Wang X., Ma H., Gu M., Lin C., Gan N., Xie Z., Wang H., Bian L., Fu L., Cai S., Chi Z., Yao W., An Z., Shi H., Huang W. (2019). Chem. Mater..

[cit8] Wang J., Huang Z., Ma X., Tian H. (2019). Angew. Chem., Int. Ed..

[cit9] Fateminia S. M. A., Mao Z., Xu S., Yang Z., Chi Z., Liu B. (2017). Angew. Chem., Int. Ed..

[cit10] Huang L., Chen B., Zhang X., Trindle C. O., Liao F., Wang Y., Miao H., Luo Y., Zhang G. (2018). Angew. Chem., Int. Ed..

[cit11] Lower S. K., El-Sayed M. A. (1966). Chem. Rev..

[cit12] Ma H., Peng Q., An Z., Huang W., Shuai Z. (2019). J. Am. Chem. Soc..

[cit13] Zhang Q., Li J., Shizu K., Huang S., Hirata S., Miyazaki H., Adachi C. (2012). J. Am. Chem. Soc..

[cit14] Zhang Q., Tsang D., Kuwabara H., Hatae Y., Li B., Takahashi T., Lee S. Y., Yasuda T., Adachi C. (2015). Adv. Mater..

[cit15] Zhao W., He Z., Lam J. W. Y., Peng Q., Ma H., Shuai Z., Bai G., Hao J., Tang B. Z. (2016). Chem.

[cit16] Yang Z., Mao Z., Zhang X., Ou D., Mu Y., Zhang Y., Zhao C., Liu S., Chi Z., Xu J., Wu Y.-C., Lu P.-Y., Lien A., Bryce M. R. (2016). Angew. Chem., Int. Ed..

[cit17] Cai S., Shi H., Li J., Gu L., Ni Y., Cheng Z., Wang S., Xiong W. W., Li L., An Z., Huang W. (2017). Adv. Mater..

[cit18] Xie Y., Ge Y., Peng Q., Li C., Li Q., Li Z. (2017). Adv. Mater..

[cit19] Hu Y., Wang Z., Jiang X., Cai X., Su S.-J., Huang F., Cao Y. (2018). Chem. Commun..

[cit20] Zhan L., Yang C., Chen Z., Gong S., Xiang Y., Ni F., Zeng X., Xie G., Yang C. (2019). Angew. Chem., Int. Ed..

[cit21] Mao Z., Yang Z., Fan Z., Ubba E., Li W., Li Y., Zhao J., Yang Z., Aldred M. P., Chi Z. (2019). Chem. Sci..

[cit22] He Z., Gao H., Zhang S., Zheng S., Wang Y., Zhao Z., Ding D., Yang B., Zhang Y., Yuan W. Z. (2019). Adv. Mater..

[cit23] Christensen P. R., Nagle J. K., Bhatti A., Wolf M. O. (2013). J. Am. Chem. Soc..

[cit24] Cruz C. D., Christensen P. R., Chronister E. L., Casanova D., Wolf M. O., Bardeen C. J. (2015). J. Am. Chem. Soc..

[cit25] Climent C., Barbatti M., Wolf M. O., Bardeen C. J., Casanova D. (2017). Chem. Sci..

[cit26] Cruz C. D., Yuan J., Climent C., Tierce N. T., Christensen P. R., Chronister E. L., Casanova D., Wolf M. O., Bardeen C. J. (2019). Chem. Sci..

[cit27] Caron É., Wolf M. O. (2017). Macromolecules.

[cit28] Brown C. M., Kitt M. J., Xu Z., Hean D., Ezhova M. B., Wolf M. O. (2017). Inorg. Chem..

[cit29] Brown C. M., Carta V., Wolf M. O. (2018). Chem. Mater..

[cit30] Caron É., Brown C. M., Hean D., Wolf M. O. (2019). Dalton Trans..

[cit31] Brown C. M., Li C., Carta V., Li W., Xu Z., Stroppa P. H. F., Samuel I. D. W., Zysman-Colman E., Wolf M. O. (2019). Inorg. Chem..

[cit32] Brown C. M., Arsenault N. E., Cross T. N. K., Hean D., Xu Z., Wolf M. O. (2020). Inorg. Chem. Front..

[cit33] Yang J., Zhen X., Wang B., Gao X., Ren Z., Wang J., Xie Y., Li J., Peng Q., Pu K., Li Z. (2018). Nat. Commun..

[cit34] Yang J., Gao H., Wang Y., Yu Y., Gong Y., Fang M., Ding D., Hu W., Tang B. Z., Li Z. (2019). Mater. Chem. Front..

[cit35] Xiong Y., Zhao Z., Zhao W., Ma H., Peng Q., He Z., Zhang X., Chen Y., He X., Lam J. W. Y., Tang B. Z. (2018). Angew. Chem., Int. Ed..

[cit36] Wang J., Chai Z., Wang J., Wang C., Han M., Liao Q., Huang A., Lin P., Li C., Li Q., Li Z. (2019). Angew. Chem., Int. Ed..

[cit37] Tian S., Ma H., Li J., Wang X., Lv A., Shi H., Geng Y., Liang F., Su Z.-M., An Z., Huang W. (2019). Angew. Chem., Int. Ed..

[cit38] Xu S., Liu T., Mu Y., Wang Y.-F., Chi Z., Lo C.-C., Liu S., Zhang Y., Lien A., Xu J. (2014). Angew. Chem., Int. Ed..

[cit39] Xu L., Zhou K., Ma H., Lv A., Pei D., Li G., Zhang Y., An Z., Li A., He G. (2020). ACS Appl. Mater. Interfaces.

[cit40] Alvarez S. (2013). Dalton Trans..

[cit41] Zhang Z., Tang L., Fan X., Wang Y., Zhang K., Sun Q., Zhang H., Xue S., Yang W. (2018). J. Mater. Chem. C.

[cit42] An Z., Zheng C., Tao Y., Chen R., Shi H., Chen T., Wang Z., Li H., Deng R., Liu X., Huang W. (2015). Nat. Mater..

[cit43] Varathan E., Subramanian V. (2017). Phys. Chem. Chem. Phys..

[cit44] Varathan E., Patnaik A. (2019). J. Phys. Chem. A.

[cit45] Turro N. J. , Ramamurthy V. and Scaiano J. C., Modern Molecular Photochemistry of Organic Molecules, University Science Books, Sausalito, CA, 1st edn, 2010

