# Supplementary material for: Controlling ultralong room temperature phosphorescence in organic compounds with sulfur oxidation state
Source: Chem Sci. 2020 Nov 2;12(1):188–95. doi: 10.1039/d0sc04715e (PMC8178747; doi:10.1039/d0sc04715e)
Supplement: SC-012-D0SC04715E-s001 [file SC-012-D0SC04715E-s001.pdf]

Electronic Supplementary Material (ESI) for Chemical Science.
This journal is © The Royal Society of Chemistry 2020

Supporting Information

Controlling Ultralong Room Temperature Phosphorescence in Organic Compounds with Sulfur Oxidation State

Zhen Xu, Clàudia Climent, Christopher M. Brown, Duane Hean, Christopher J. Bardeen,* David Casanova* and Michael O. Wolf*

General

Solvents used were reagent grade and used without any further purification. HPLC grade solvents were used for analyses. 9-H carbazole was purchased from Sigma-Aldrich (China) and was crystallized from toluene solution before use. Other purchased chemicals were used without further purification. Bis(4-bromophenyl)sulfane was synthesized following the literature procedure.[1]

Spectroscopy

^1H, $^{13}C\{^1H\}$, COSY, NOESY, HSQC and HMBC NMR experiments were collected using a Bruker AV-400 spectrometer and referenced first to TMS and then to the residual protonated solvent peak. NMR solvents (Aldrich or Cambridge Isotope Laboratories) were used as received. Electrospray ionization mass spectrometry data were obtained using a Bruker Esquire LC ion trap mass spectrometer. Infrared spectroscopy was performed on an attenuated total reflection (ATR) crystal using a Perkin-Elmer Frontier FT-IR spectrometer. UV-vis absorption spectra were recorded on a Varian-Cary 5000 UV-Vis-near-IR spectrophotometer. Steady-state photoluminescence data were collected using a Photon Technology International (PTI) QuantaMaster 50 fluorimeter fitted with an integrating sphere, double excitation monochrometer and utilizing a 75 W Xe arc lamp as the source. Fluorescence and phosphorescence lifetime data were collected using a Horiba Yvon Fluorocube TCSPC apparatus with a 370 nm NanoLED (fluorescence) or a 359 nm Horiba spectral LED (phosphorescence). The photoluminescence lifetime data were fitted using the DAS6 Data Analysis software package. Time-resolved photoluminescence spectra utilizing a 1 ms delay were measured using a Photon Technology International (PTI) QuantaMaster 400 equipped with a 359 nm Horiba spectral LED. Time-resolved spectra ranging from microsecond to 110

ms were measured using the same spectrometer coupled with a 320 nm Ng:YAG laser. Samples for low-temperature spectroscopy and lifetime measurements were cooled using an Oxford Instruments Optistat DN coupled with an ITC601 temperature controller.

X-Ray Diffraction

Single-crystal X-ray data were collected using a Bruker APEX DUO diffractometer with graphite monochromated Mo Kα radiation (λ = 0.71073 Å) at 100 K. Raw frame data were processed using APEX2. The program SAINT+, v.7.68 was used to reduce the data and the program SADABS was used to make corrections to the empirical absorptions. Space group assignments were made using XPREP on all compounds. In all cases, the structures were solved in the Olex2 suite[2] of programs using Intrinsic Phasing and refined using full-matrix least-squares/difference Fourier techniques on F2 using SHELXL.[3] Diagrams and publication material were generated using CrystalMaker. Powder X-ray crystallography data were collected on a Bruker X8 APEX II diffractometer with graphite monochromated Mo-Kα radiation.

Photophysical Data

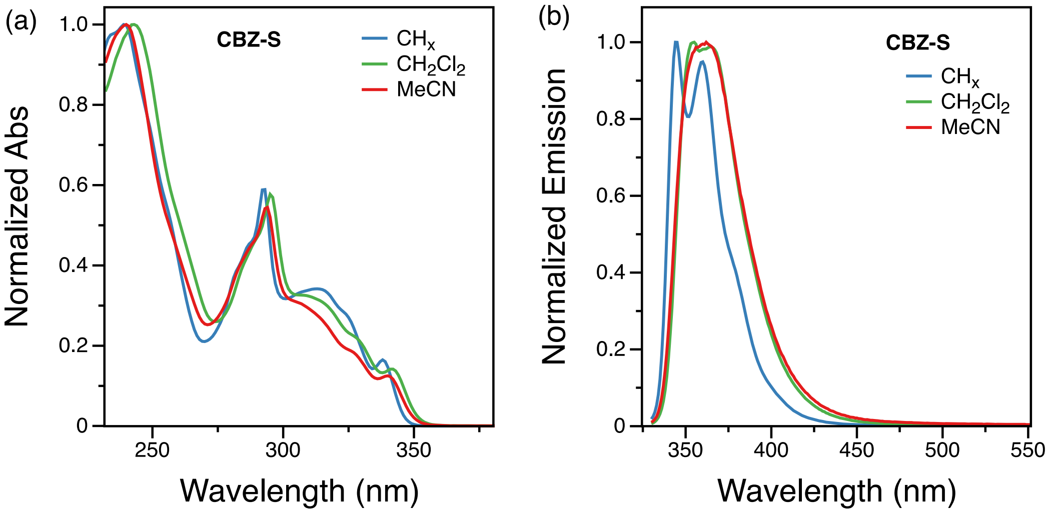

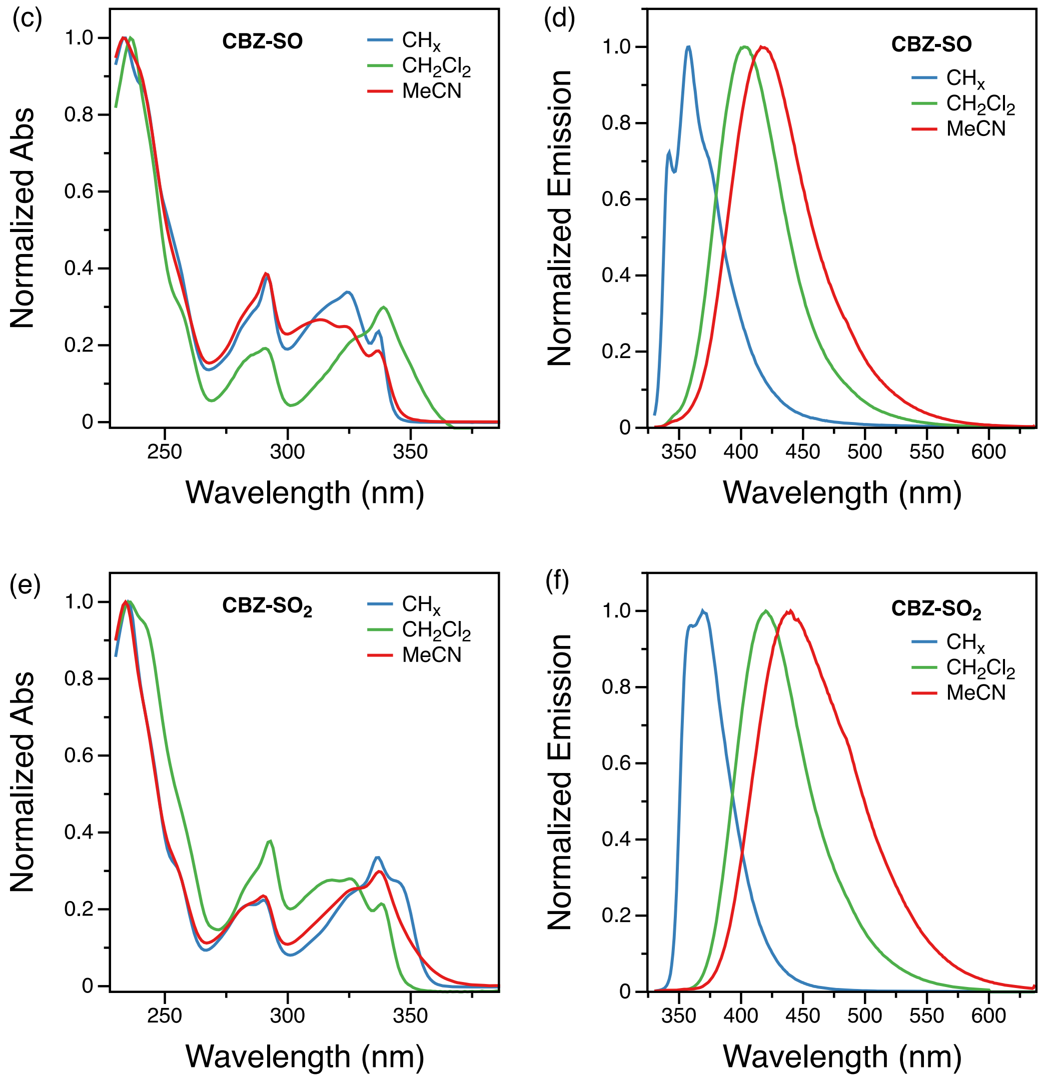

Figure S1. Normalized absorption spectra of (a) **CBZ-S**, (c) **CBZ -SO** and (e) **Cbz CBZ -SO$_2$**. Normalized photoluminescence spectra of (b) **CBZ-S**, (d) **CBZ -SO** and (f) **CBZ-SO$_2$**. (λ_{ex} = 320 nm). All spectra were recorded in ~ 2 × 10^{-5} M cyclohexanes (CH_x), dichloromethane (CH_2Cl_2) and acetonitrile (MeCN) solutions at room temperature.

RTP Arbitrary Efficiency Φ:

$$\Phi = A_P \frac{\Phi_F}{A_F}$$

$A_{P:}$ Integration of phosphorescence peak

$A_{F:}$ Integration of fluorescence peak

Φ_F: fluorescence quantum yield.

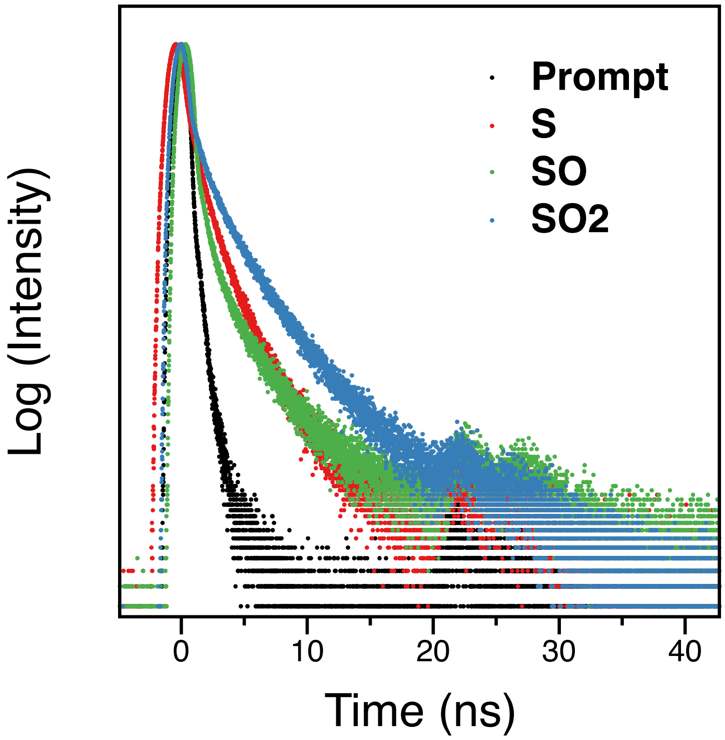

Figure S2. Fluorescence decay profile of **CBZ-S, CBZ-SO** and **CBZ-SO_2** in the crystalline state.

Table S1. Summary of solid state photophysical data

	PLQY	*Fluorescence*	*Phosphorescence (Air)*[b]			*Phosphorescence (77K)* [b]	
		λ_{em} *(nm)*[a]	λ_{em} *(nm)*	τ *(ms)* [c]	τ_{avg} *(ms)* [d]	τ *(ms)* [c]	τ_{avg} *(ms)* [d]
**CBZ-S**	0.27	390	565	29.84 (0.37),<br>169.13 (0.48),<br>373.46 (0.15)	226.97	245.26(0.30),<br>777.19 (0.54),<br>1657.3 (0.16)	1033.1
**CBZ-SO**	0.21	388	565	27.05 (0.26),<br>114.92 (0.55),<br>445.02 (0.19)	291.24	245.26(0.17),<br>808.92 (0.51),<br>1877.4 (0.32)	1395.1
**CBZ-SO_2**	0.75	388	562	24.28 (0.24),<br>98.87 (0.58),<br>471.37 (0.18)	309.46	288.89 (0.08),<br>1173.8(0.62),<br>2461.8 (0.30)	1798.7

[a] λ_{ex} = 320 nm. [b] λ_{ex} = 359 nm. [c] Relative amplitudes in the parenthesis. [c] $\tau_{avg} = \frac{\sum A^2\tau}{\sum A\tau}$ (A: relative amplitude)

Powder X-Ray Diffraction Data

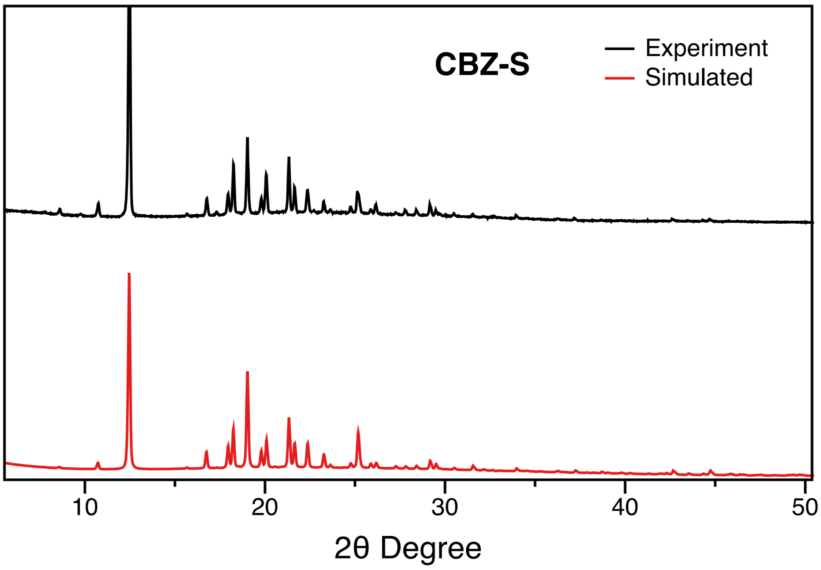

Figure S3. Powder X-ray diffraction (pXRD) patterns of **CBZ-S** crystalline powder (black) compared to their single crystal simulated patterns (red).

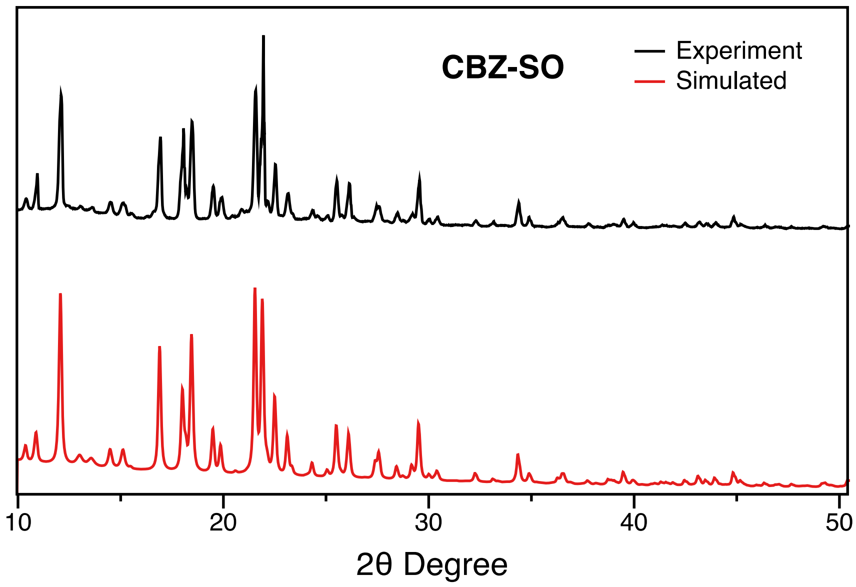

Figure S4. Powder X-ray diffraction (pXRD) patterns of **CBZ-SO** crystalline powder (black) compared to their single crystal simulated patterns (red).

Synthetic Details

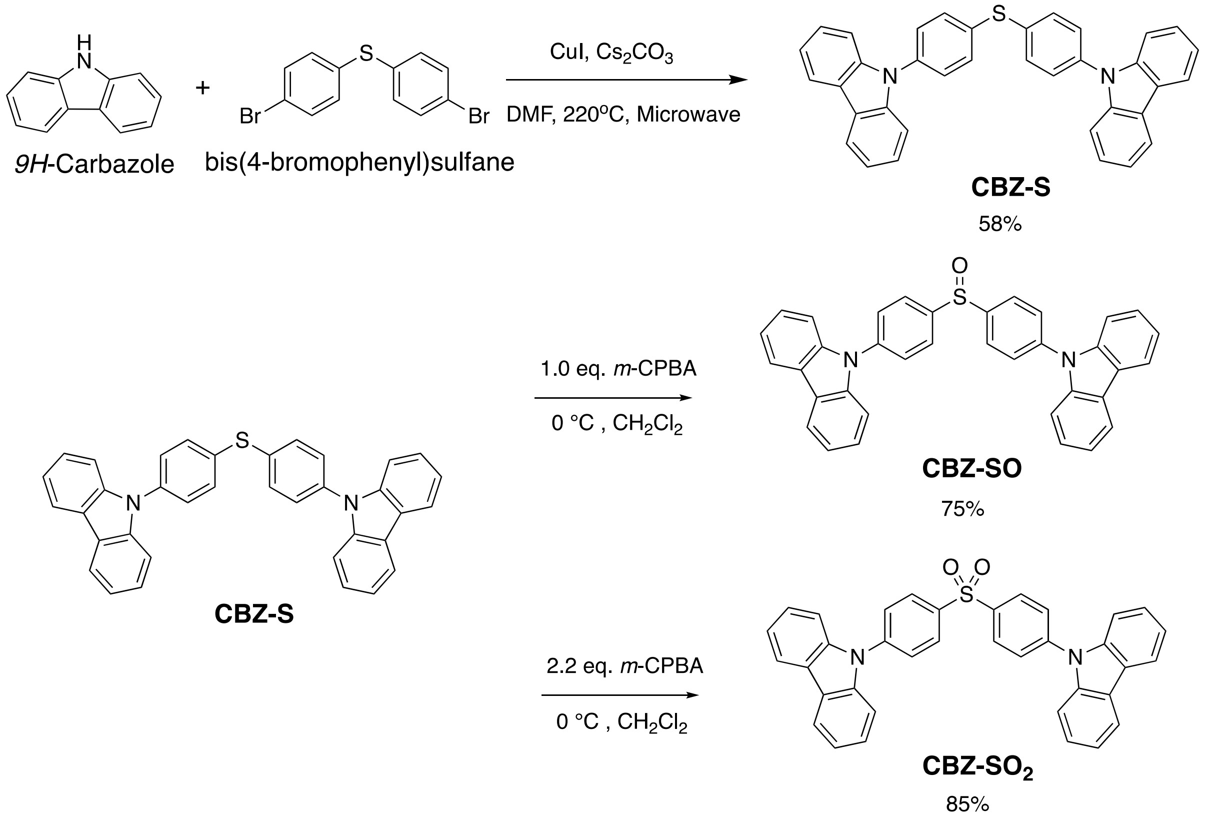

Scheme S1. Synthetic route of **CBZ-S, CBZ-SO** and **CBZ-SO$_2$**.

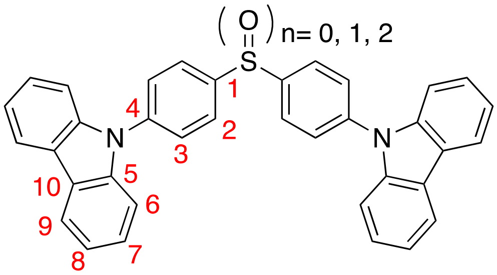

CBZ-S

This compound has been previously synthesized.[4] Bis(4-bromophenyl)sulfane (344 mg, 1.0 mmol, 1.0 equiv.), *9H*-Carbazole (334 mg, 2.0 mmol, 2.0 equiv.), Cs_2CO_3 (650 mg, 2.0 mmol, 2.0 equiv.), CuI (38 mg, 0.2 mmol, 0.2 equiv.) and DMF (2 mL) were added to a 5-mL vial. The vial was sealed and placed in a Biotage microwave cavity. After irradiation at 220 °C for 80 minutes, the reaction mixture was poured into water (25 mL) and extracted with CH_2Cl_2 (3 × 15 mL). The organic layers were combined and dried over anhydrous $MgSO_4$, filtered, and concentrated under vacuum. Purification by column chromatography (1:30 EtOAc: hexanes) gave the pure product as a white solid (300 mg, 0.58 mmol, 58%). ^1H NMR (CD_2Cl_2, 400 MHz): δ = 8.16 (H_9, d, *J* = 7.7 Hz, 4 H), 7.71 (H_2, d, *J* = 8.5 Hz, 4 H), 7.61 (H_3,d, *J* = 8.5 Hz, 4 H), 7.48 (d, *J* = 7.3 Hz, 4 H), 7.43(t, *J* = 7.5 Hz, 4 H), 7.30 (H_8, t, 7.3 Hz, 4 H). $^{13}C\{^1H\}$ NMR (CD_2Cl_2, 101 MHz): δ 141.3 (C_5), 137.5 (C_4), 135.1 (C_1), 133.12 (C_2), 128.4 (C_3), 126.6 (C_7), 124.0 (C_{10}), 120.8 (C_9), 120.7 (C_8), 110.3 (C_6). HRMS: calculated m/z: 516.1660, found (EI) m/z: 516.1658 $[M]^+$.

CBZ-SO

To a solution of **CBZ-S** (0.103 g, 0.20 mmol, 1.0 equiv.) in CH_2Cl_2 (10 mL) at 0 °C was added 70% *m*-chloroperoxybenzoic acid (*m*-CPBA, 0.050 g, 0.20 mmol, 1.0 equiv.) and the mixture was stirred for 0.5 h. The reaction mixture was poured into a saturated aqueous solution of sodium bicarbonate (15 mL) and was then extracted with CH_2Cl_2 (3 × 10 mL). The organic layers were combined, dried over anhydrous $MgSO_4$, filtered, and concentrated under vacuum. Purification by column chromatography (CH_2Cl_2) gave the pure product as a white solid (0.080 g, 0.15 mmol, 75%). ^1H NMR (CD_2Cl_2, 400 MHz): δ = 8.15 (H_9, d, *J* = 7.7 Hz, 4 H), 8.01 (H_2, d, *J* = 7.7 Hz, 4 H), 7.80 (H_3, d, *J* = 7.8 Hz, 4 H), 7.49 (H_6, d, *J* = 8.1 Hz, 4 H), 7.43 (H_7, t, *J* = 7.6 Hz, 4 H), 7.31 (H_8, t, *J* = 7.4 Hz, 4 H). $^{13}C\{^1H\}$ NMR (CD_2Cl_2, 101 MHz): δ 144.9 (C_1), 141.1 (C_4), 141.0 (C_5), 128.3 (C_3), 127.1 (C_2), 126.8 (C_7), 124.3 (C_{10}), 121.1 (C_8), 120.9 (C_9),

110.2 (C_6). IR (neat): (σ (SO)) 1042 cm^{-1}. HRMS: calculated m/z: 532.1609, found (EI) m/z: 533.1686 $[M+H]^+$.

CBZ-SO_2

This compound has been previously synthesized.[5] To a solution of **CBZ-S** (0.103 g, 0.20 mmol) in CH_2Cl_2 (4 mL) at 0 °C was added 70% *m*-chloroperoxybenzoic acid (*m*-CPBA, 0.108 g, 0.44 mmol, 2.2 equiv.) and the mixture was stirred for 1 hour. The reaction mixture was poured over a saturated aqueous solution of sodium bicarbonate (20 mL) and extracted with CH_2Cl_2 (3 × 15 mL). The organic layers were combined, dried over anhydrous $MgSO_4$, filtered, and concentrated under vacuum. The crude material was purified by column chromatography (2:1 hexanes: CH_2Cl_2) to afford the pure product as a white solid (93 mg, 0.17 mmol, 85%). ^{1}H NMR (CD_2Cl_2, 400 MHz): δ = 8.28 (H_2, d, *J* = 8.6 Hz, 4 H), 8.15 (H_9, d, *J* = 7.7 Hz, 4 H), 7.85 (H_3, d, *J* = 8.6 Hz, 4 H), 7.52 (H_6, d, *J* = 8.2 Hz, 4 H), 7.43 (H_7, t, *J* = 7.7 Hz, 4 H), 7.33 (H_8, t, *J* = 7.5 Hz, 4 H). $^{13}C\{^1H\}$ NMR (CD_2Cl_2, 101 MHz): δ 143.2 (C_4), 140.6 (C_5), 140.1 (C_1), 130.3 (C_2), 127.8 (C_3), 126.9 (C_7), 124.7 (C_{10}), 121.5 (C_8), 120.0 (C_9), 110.2 (C_6). IR (neat): (σ (SO_2)) 1154 and 1313 cm^{-1}.

NMR Spectra

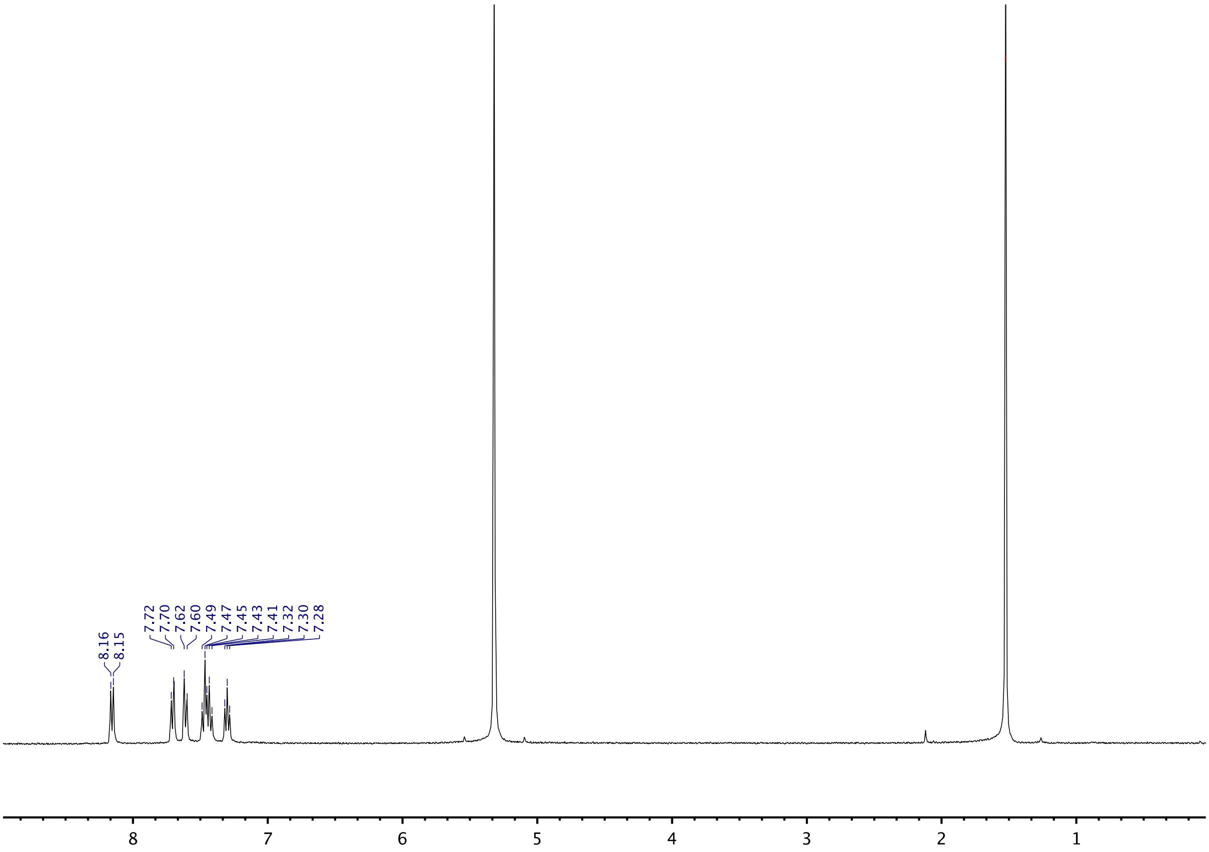

Figure S5. ^{1}H NMR spectra of **CBZ-S** in CD_2Cl_2 at 25 ^{o}C.

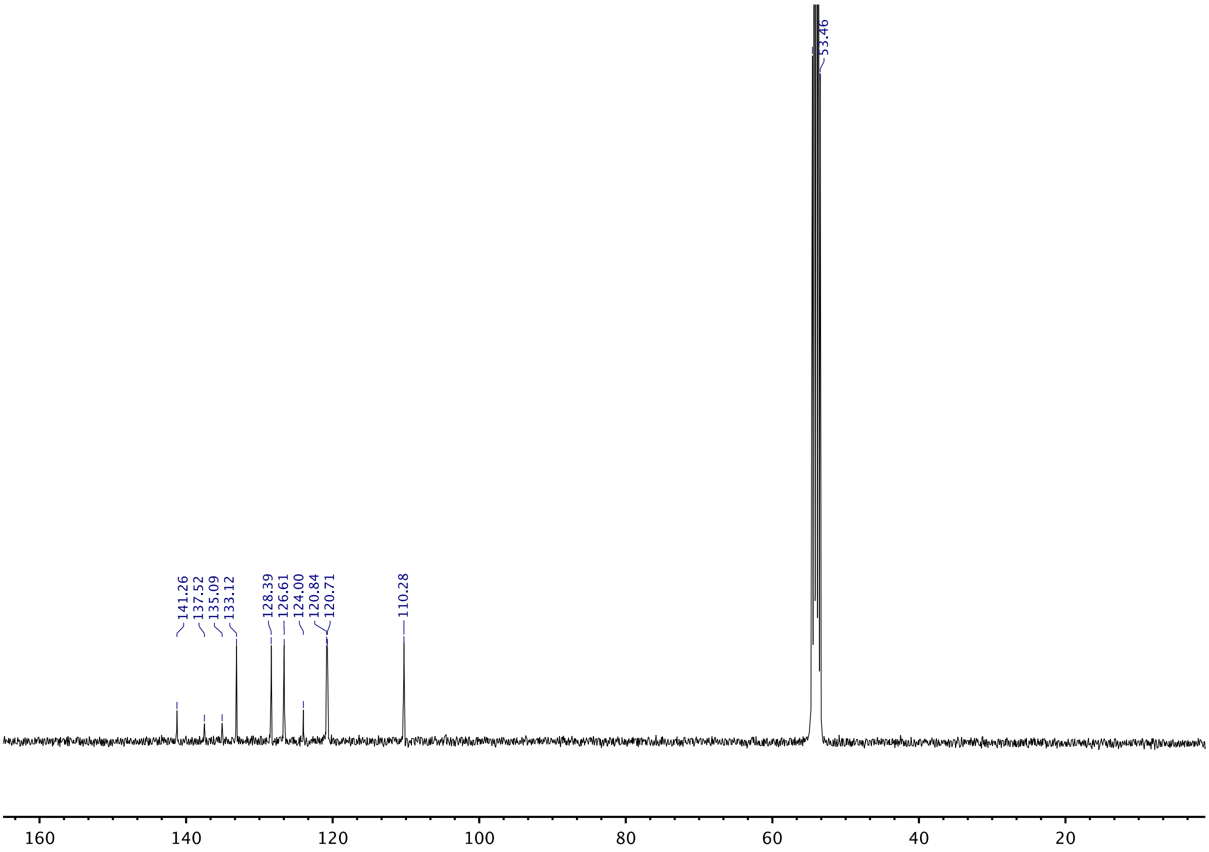

Figure S6. $^{13}C\{^{1}H\}$ NMR spectra of **CBZ-S** in CD_2Cl_2 at 25 ^{o}C.

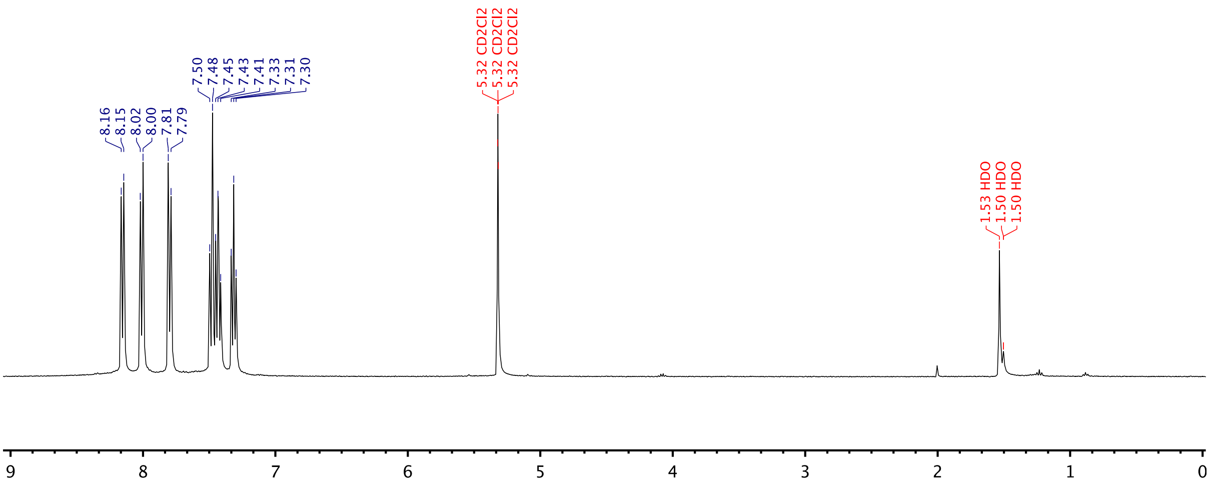

Figure S7. ^{1}H NMR spectra of **CBZ-SO** in CD_2Cl_2 at 25 °C.

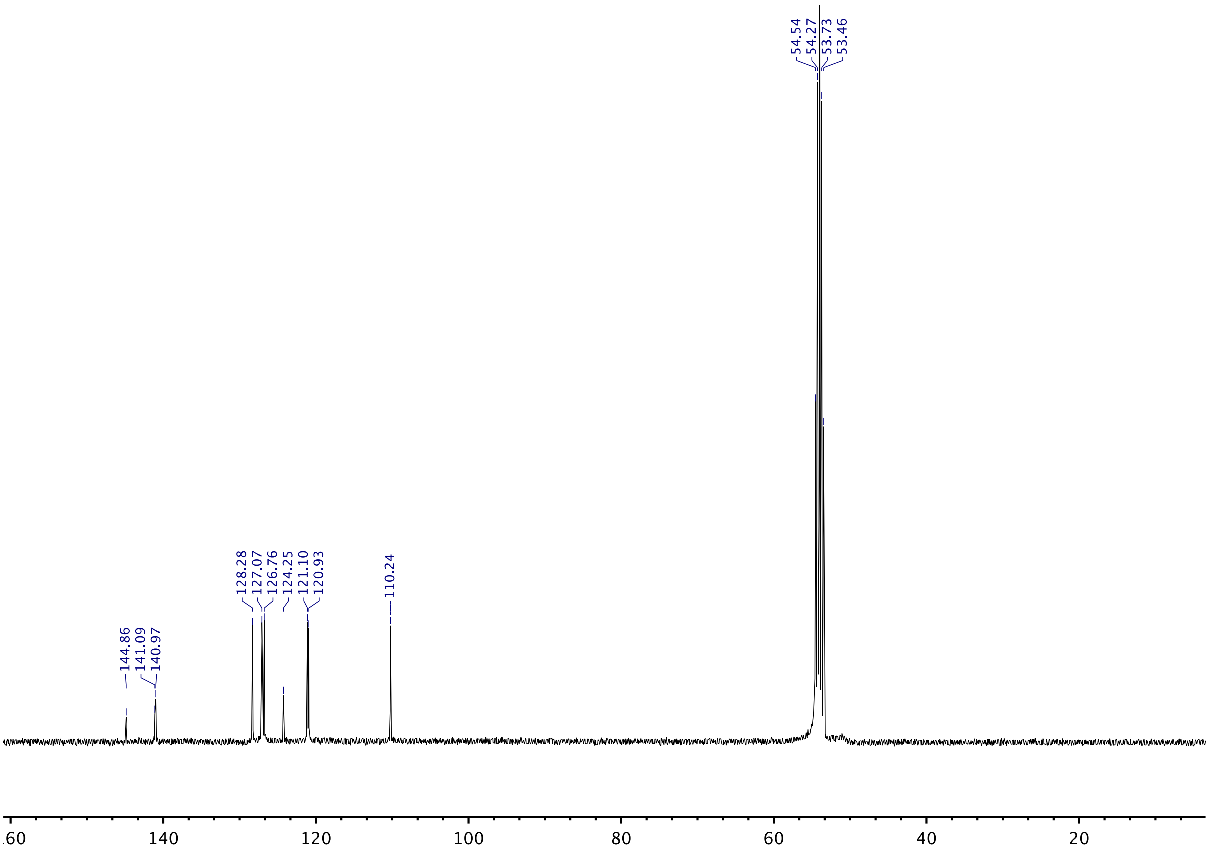

Figure S8. ^{13}C{^{1}H} NMR spectra of **CBZ-SO** in CD_2Cl_2 at 25 °C.

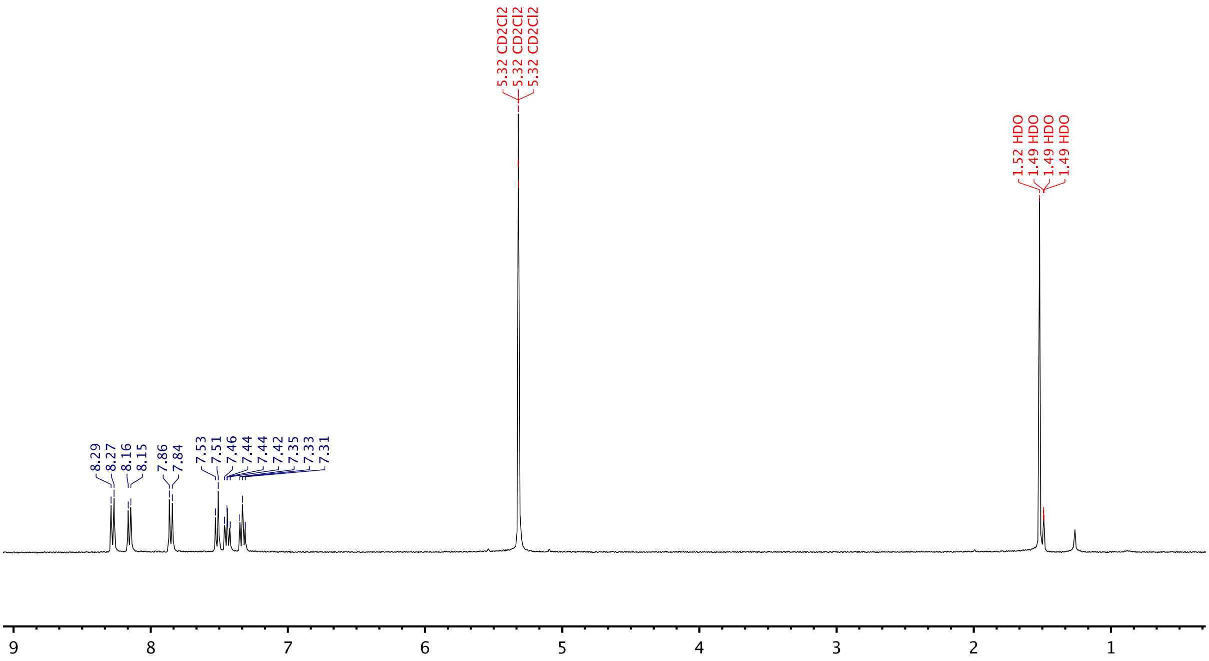

Figure S9. ^{1}H NMR spectra of **CBZ-SO_2** in CD_2Cl_2 at 25 °C.

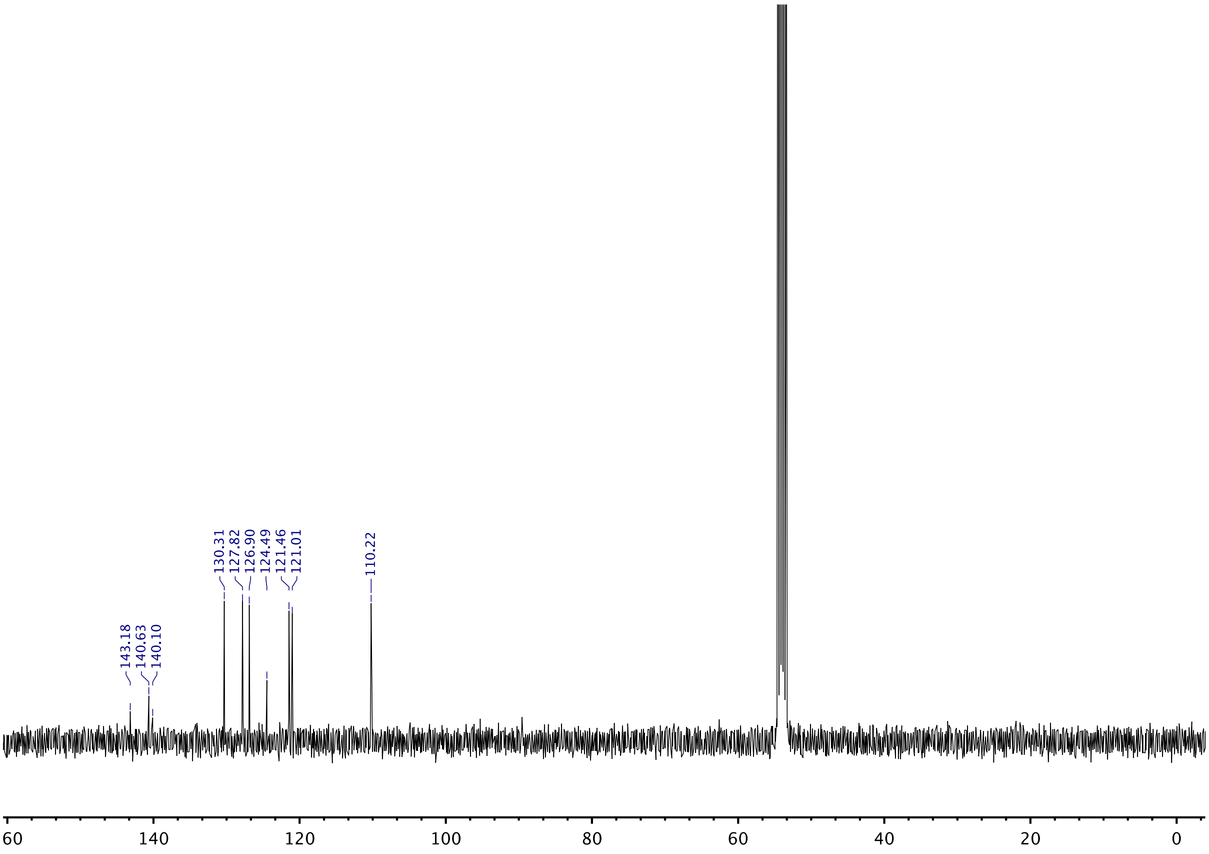

Figure S10. ^{13}C{^{1}H} NMR spectra of **CBZ-SO_2** in CD_2Cl_2 at 25 °C.

Computational Details

Molecular geometries have been optimized within the density functional theory (DFT) with the ωB97X-D exchange-correlation functional[6] and the 6-31+G(d) atomic basis set. Electronic transitions to the lowest singlet and triplet states and the interstate (singlet-triplet) spin-orbit couplings have been computed with the time-dependent version of DFT (TDDFT), and with the same energy functional and basis set. All calculations have been done with the Q-Chem package.[7]

Table S2. Bond angles C-S-C and C-S-O, and dihedral angles Ph-CBZ, Ph-Ph and CBZ-CBZ (in degrees) for the crystal and optimized molecular structures of the **CBZ-SO$_n$** dimers. C-S-O angles in **CBZ-SO$_2$** correspond to average values.

	crystal			optimized		
angle	**CBZ-S**	**CBZ-SO**	**CBZ-SO$_2$**	**CBZ-S**	**CBZ-SO**	**CBZ-SO$_2$**
C-S-C	104.8	100.3	106.2	101.8	95.8	103.2
C-S-O	-	103.9	107.8	-	106.5	107.6
Ph-CBZ	118.5	125.4	123.6	122.2	123.5	125.6
Ph-Ph	82.5	69.1	70.2	66.6	9.9	2.6
CBZ-CBZ	2.1	4.1	4.1	23.5	77.7	93.1

Table S3. Vertical excitation energies (in eV), oscillator strengths (*f* in parenthesis) and electronic character to the lowest singlet and triplet states of the 9-phenylcarbazole molecule computed at the ground state optimized geometry at the ωB97X-D/6-31+G(d) level. CBZ/Ph = local excitation on carbazole/phenyl; CT = CBZ→Ph charge transfer.

state	ΔE (*f*)	character
T_1	3.25	CBZ (B_2)
T_2	3.56	CBZ (A_1)
T_3	3.66	Ph; CT
S_1	4.38 (0.050)	CBZ (A_1); CT
S_2	4.82 (0.164)	CBZ (B_2)
S_3	4.91 (0.156)	CT

Table S4. Vertical excitation energies (in eV), oscillator strengths (*f* in parenthesis), electronic character, and orbital contributions (in %) to the lowest singlet and triplet states of the **CBZ-SO$_n$** dimers computed at the ground state optimized geometries at the ωB97X-D/6-31+G(d) level. CBZ/Ph = local excitation on carbazole/phenyl; CT = CBZ→Ph charge transfer; n(S): lone-pairs of S atom. H = HOMO; L = LUMO.

state	ΔE (f)	character	% contribution
**CBZ-S**			
T_1	3.24	CBZ	16,16 H-2→L+1,L+2; 15,15 H-3→L+1,L+2
T_2	3.24	CBZ	16,16 H-3→L+1,L+2; 15,15 H-2→L+1,L+2
T_3	3.35	Ph; CT; n(S)→Ph	29,24 H,H-4→L; 9 H-7→L+3
T_4	3.42	Ph; CT; n(S)→Ph	22,18 H,H-4→L+4; 12,10 H-8,H-1→L
S_1	4.38 (0.219)	CBZ; n(S)→CZ	38 H→L+1; 38 H-1→L+2
S_2	4.39 (0.034)	CBZ; n(S)→CZ	42 H-1→L+1; 40 H→L+2
S_3	4.47 (0.486)	CT; n(S)→Ph	59 H→L; 15 H-1→L+4; 11 H-4→L
**CBZ-SO**			
T_1	3.24	CBZ	57 H-2→L+3; 7 H→L+8
T_2	3.24	CBZ	57 H-3→L+2; 9 H-1→L+7
T_3	3.47	Ph; CT	23,16 H-7,H→L; 8 H-8→L+1
T_4	3.51	Ph; CT; n(S,O)→Ph	13,10 H-1,H-4→L; 10,10 H,H-7→L+4
S_1	4.40 (0.163)	CBZ	40 H→L+3; 20,12 H-1→L+2,L+3
S_2	4.41 (0.051)	CBZ	44,9 H-1,H→L+2; 26 H→L+3
S_3	4.48 (0.331)	CT	47 H→L; 17,11 H-1→L+4,L; 12 H-6→L+4
**CBZ-SO$_2$**			
T_1	3.24	CBZ	31 H-3→L+3; 31 H-2→L+2
T_2	3.24	CBZ	31 H-3→L+2; 31 H-2→L+3
T_3	3.37	Ph; CT	30,26 H,H-2→L; 10 H-1→L+4
T_4	3.51	Ph; CT	21 H-1→L; 17 H→L+4
S_1	4.27 (0.511)	CT	68 H→L; 22 H-1→L+4
S_2	4.42 (0.174)	mainly CT; CBZ	43,13 H-1→L,L+2; 19,13 H→L+4,L+3
S_3	4.43 (0.026)	CBZ	42 H→L+2; 41 H-1→L+3

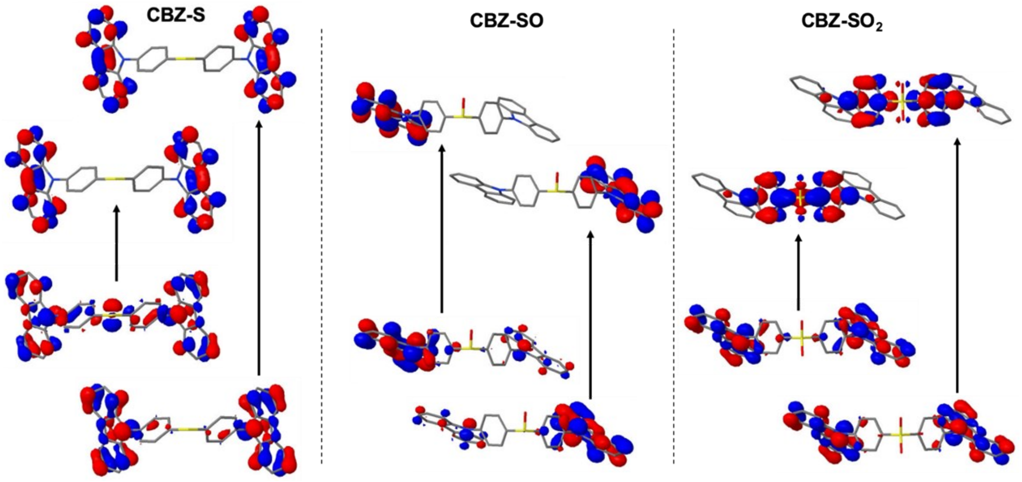

Figure S11. Main orbital contributions to the S_1 excitation of **CBZ-SO$_n$** at the Franck-Condon geometry computed at the ωB97X-D/6-31+G(d) level. Note: the LUMO of **CBZ-SO$_2$** shows overlap of π-orbitals of the two carbons bonded to S (but no contributions from S orbitals). The weight of O atomic orbitals in the LUMO is very small.

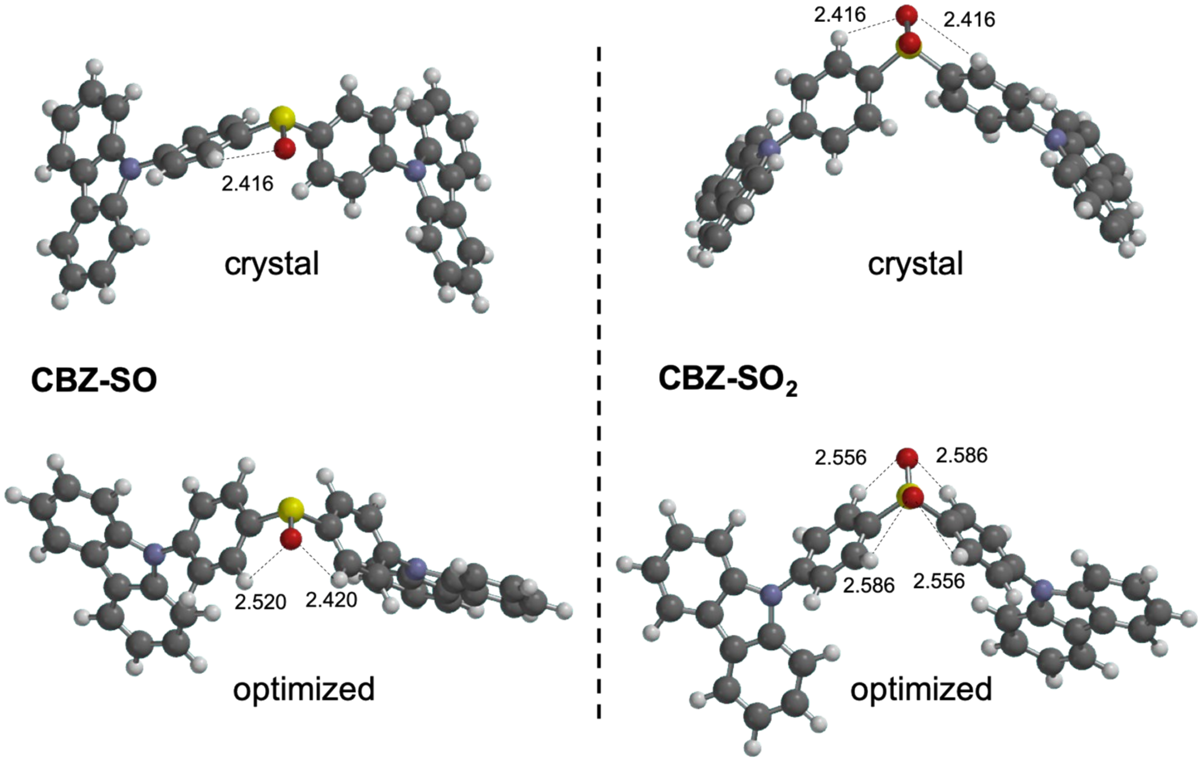

Figure S12. Crystal (top) and optimized (bottom) molecular structures of **CBZ-SO** and **CBZ-SO$_2$** dimers. Short SO···H contacts (dashed lines) indicated in Angstroms.

Table S5. Vertical deexcitation energies (in eV), oscillator strengths (*f* in parenthesis) and electronic character from the optimized singlet and triplet excited state potential energy surface of the **CBZ-SO$_n$** dimers computed at the ωB97X-D/6-31+G(d) level. S_{loc} = localized singlet, S_{deloc} = delocalized singlet, S_{ex} = excimer-like, T_1 = local triplet.

molecule	state	ΔE (*f*)
**CBZ-S**	S_{loc}	4.13 (0.086)
	S_{deloc}	3.85 (0.569)
	T_1	2.47
**CBZ-SO**	S_{loc}	4.17 (0.084)
	S_{ex}	3.64 (0.075)
	T_1	2.47
**CBZ-SO$_2$**	S_{ex}	3.50 (0.060)
	T_1	2.47

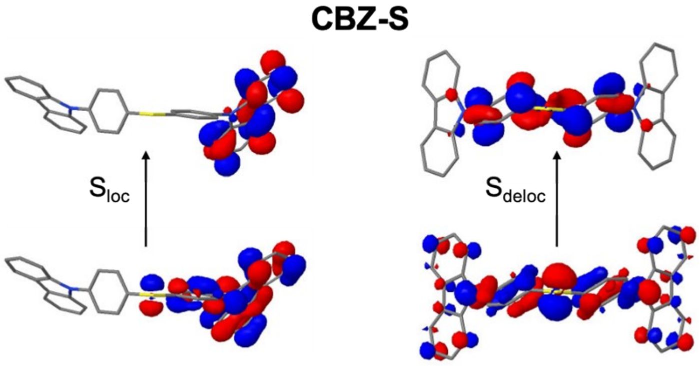

Figure S13. Main orbital contributions to the local (S_{loc}, left) and delocalized (S_{deloc}, right) excited state minima of **CBZ-S** computed at the ωB97X-D/6-31+G(d) level.

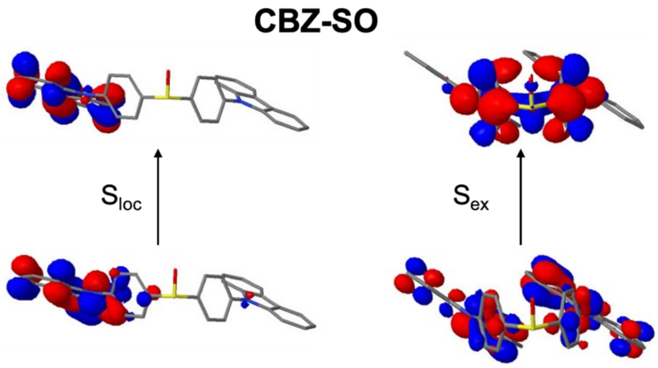

Figure S14. Main orbital contributions to the local (S_{loc}, left) and excimer-like (S_{ex}, right) excited state minima of **CBZ-SO** computed at the ωB97X-D/6-31+G(d) level.

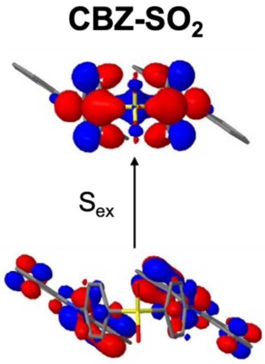

Figure S15. Main orbital contributions to the excimer-like (S_{ex}) state minimum of **CBZ-SO$_2$** computed at the ωB97X-D/6-31+G(d) level.

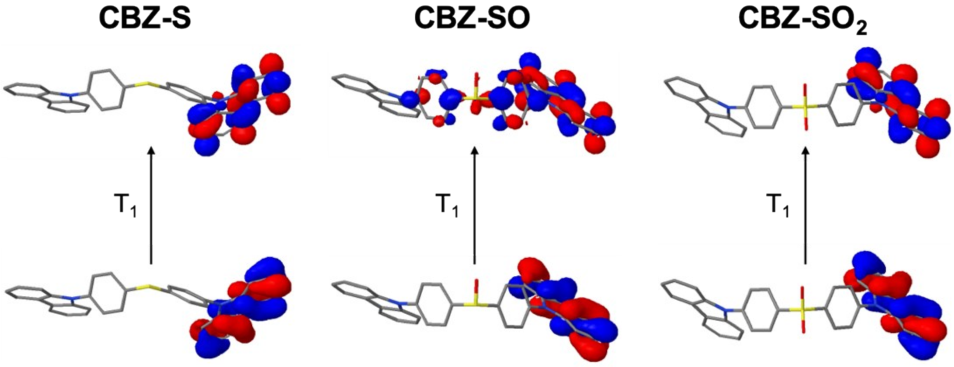

Figure S16. Main orbital contributions to the lowest triplet (T_1) state minimum of **CBZ-SO$_n$** computed at the ωB97X-D/6-31+G(d) level.

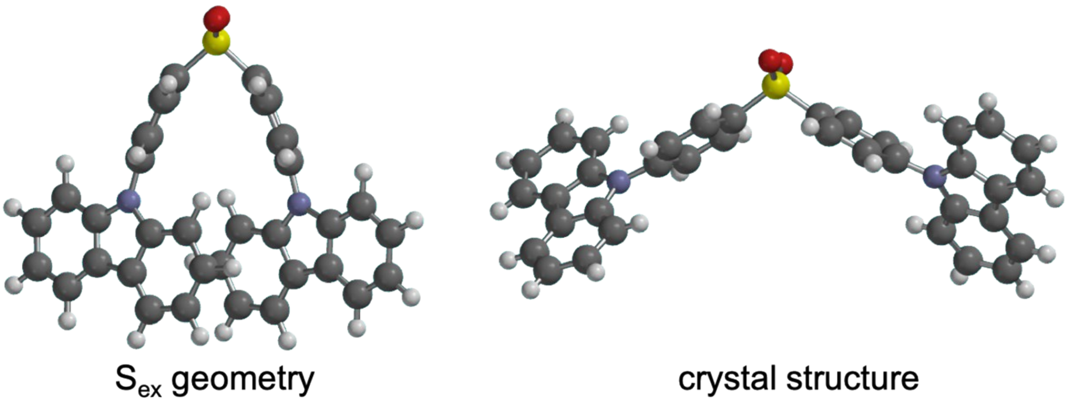

Figure S17. Excited state (S_{ex}) optimized geometry and crystal molecular structure of **CBZ-SO$_2$**.

Table S6. Vertical excitation energies (in eV), oscillator strengths (*f* in parenthesis) and electronic character to the lowest singlet and triplet states of the **CBZ-SO$_n$** dimers for the crystal molecular geometries computed at the ωB97X-D/6-31+G(d) level. CBZ/Ph = local excitation on carbazole/phenyl; CT = CBZ→Ph charge transfer.

		ΔE (*f*)		
state	**CBZ-S**	**CBZ-SO**	**CBZ-SO$_2$**	character
T_1	3.21	3.20	3.27	CBZ; CT
T_2	3.21	3.21	3.27	CBZ; CT
T_3	3.37[a]	3.42	3.52	CBZ; Ph; CT
T_4	3.48[a]	3.48	3.53	CBZ; Ph; CT
S_1	4.34 (0.306)[a]	4.35 (0.507)[a]	4.44 (0.490)[b]	CBZ; Ph; CT
S_2	4.37 (0.068)[a]	4.41 (0.089)	4.48 (0.115)	CBZ; Ph; CT
S_3	4.48 (0.487)[a]	4.45 (0.013)[a]	4.55 (0.200)	CBZ; Ph; CT

[a] n(S)→Ph (also); [b] CBZ→Ph (mainly).

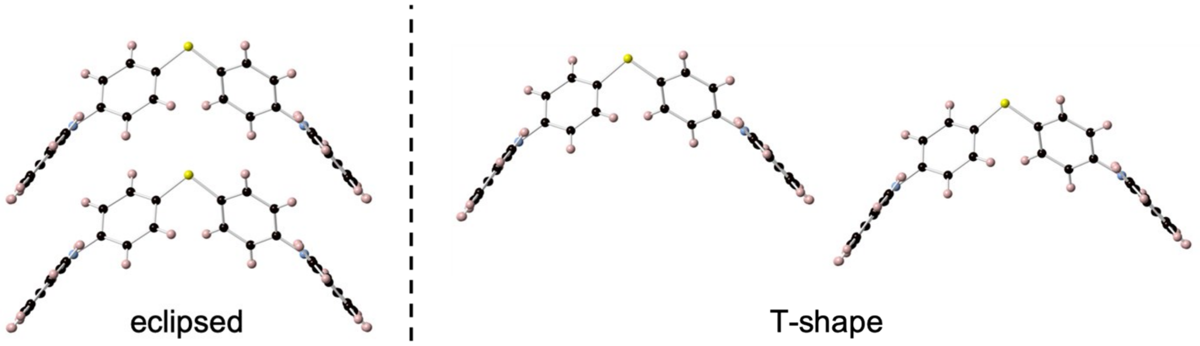

Figure S18. Crystal molecular dimers with the shortest intermolecular distance. Eclipsed (left) and T-shape (right) molecular pairs of **CBZ-SO** in the crystal. **CBZ-SO** and **CBZ-SO$_2$** crystals exhibit equivalent molecular pairs.

Table S7. Vertical excitation energies (in eV), oscillator strengths (*f* in parenthesis) to the lowest singlet and triplet states of computed for the eclipsed and T-shape molecular pairs of **CBZ-SO$_n$** in the crystal (Figure S17) computed at the ωB97X-D/6-31+G(d) level.

state	**CBZ-S**	**CBZ-SO**	**CBZ-SO$_2$**
eclipsed pair			
T_1	3.24	3.23	3.29
T_2	3.24	3.24	3.29
T_3	3.24	3.24	3.29
T_4	3.28	3.25	3.30
S_1	4.32 (0.166)	4.34 (0.022)	4.41 (0.009)
S_2	4.33 (0.150)	4.37 (0.451)	4.43 (0.062)
S_3	4.34 (0.135)	4.37 (0.144)	4.44 (0.503)
S_4	4.35 (0.010)	4.40 (0.134)	4.48 (0.143)
T-shape pair			
T_1	3.22	3.22	3.28
T_2	3.23	3.23	3.30
T_3	3.24	3.23	3.30
T_4	3.25	3.25	3.31
S_1	4.32 (0.664)	4.34 (1.144)	4.41 (1.117)
S_2	4.35 (0.118)	4.36 (0.005)	4.45 (0.001)
S_3	4.36 (0.093)	4.41 (0.017)	4.48 (0.117)
S_4	4.38 (0.100)	4.42 (0.171)	4.49 (0.177)

Table S8. Relative energies (in meV) and SOCs (in cm^{-1}) between the lowest excited singlet (S_1) and energetically close triplets of the **CBZ-SO$_n$** dimers computed at the ωB97X-D/6-31+G(d) level in the crystal molecular structure. The last column qualitatively indicates the weight of the sulfur and oxygen lone pairs participation in the electronic structure of T_n.

molecule	state	$E(S_1)$-$E(T_n)$	SOC	n(S) or n(O) character
**CBZ-S**	T_7	211	0.8	large
	T_8	163	0.6	small
	T_9	162	0.2	small
	T_{10}	82	0.8	small
	T_{11}	79	0.3	small
	T_{12}	34	0.4	large
	T_{13}	-281	0.0	small
	T_{14}	-313	0.8	small
	T_{15}	-376	0.2	small
	T_{16}	-377	0.5	small
	T_{17}	-432	0.7	small
	T_{18}	-433	0.5	small
	T_{19}	-605	3.1	large
	T_{20}	-642	0.2	small
**CBZ-SO**	T_7	360	8.6	large
	T_8	182	3.6	small n(s), n(O)
	T_9	175	0.8	no
	T_{10}	93	0.9	no
	T_{11}	85	0.4	no
	T_{12}	-3	3.3	small n(O)
	T_{13}	-70	2.7	small n(S), n(O)
	T_{14}	-196	5.6	small n(S), n(O)
	T_{15}	-253	2.5	small n(S), n(O)
	T_{16}	-328	3.2	small n(S), n(O)
	T_{17}	-334	1.5	small n(S)
	T_{18}	-369	4.1	small n(O)
	T_{19}	-394	0.4	no
	T_{20}	-472	36.9	large n(O), n(S)
**CBZ-SO$_2$**	T_7	197	0.2	no
	T_8	197	0.5	no
	T_9	121	1.0	no
	T_{10}	120	0.1	no
	T_{11}	-31	0.3	no
	T_{12}	-47	0.3	no
	T_{13}	-204	0.4	no
	T_{14}	-213	0.3	no
	T_{15}	-297	0.4	no
	T_{16}	-297	0.3	no
	T_{17}	-339	0.4	no
	T_{18}	-339	0.1	no
	T_{19}	-477	0.3	small n(O)
	T_{20}	-490	0.4	small n(O)

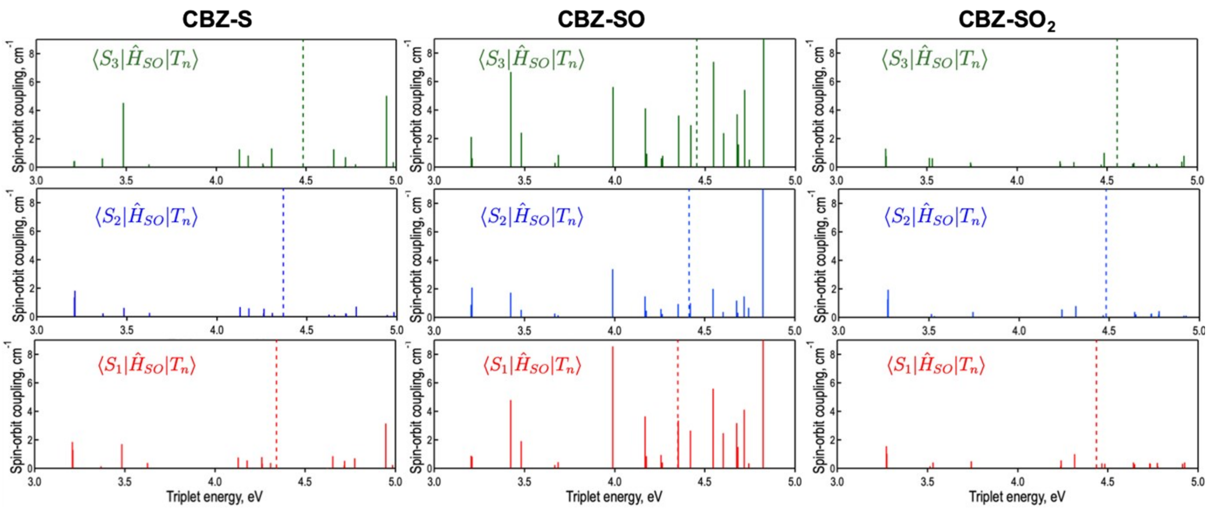

Figure S19. Spin orbit couplings (in cm^{-1}) between excited triplet states and S_1 (red), S_2 (blue) and S_3 (green) of **CBZ-SO$_n$** for the crystal molecular structure calculated at the ωB97X-D/6-31+G(d) level. Dashed vertical lines indicate the energy of the excited singlet, S_1, S_2 or S_3, respectively.

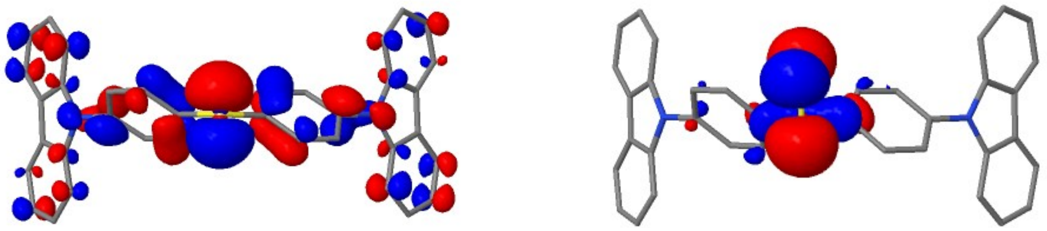

Figure S20. Molecular orbital sulfur lone pair of **CBZ-S** (left) and **CBZ-SO** (right) responsible for the strong S_1/T_n SOCs in the crystal molecular structure calculated at the ωB97X-D/6-31+G(d) level.

Phosphorescent emission probability

We evaluate the oscillator strength of triplet emission through perturbation theory by expanding the triplet state wave function as:

$$|T_1^{SOC}\rangle = |T_1\rangle + \sum_n \frac{\langle S_n|\hat{H}_{SO}|T_1\rangle}{E(T_1) - E(S_n)}|S_n\rangle \quad . \tag{S1}$$

Then, the oscillator strength (f) can be expressed as:

$$f = \frac{2}{3}\Delta E_{ST}|\langle T_1^{SOC}|\boldsymbol{\hat{\mu}}|S_0\rangle|^2 \tag{S2}$$

Table S9. Oscillator strengths (f) for the emission from the two lowest excited triplet states of **CBZ-SO$_n$** computed with equation S2 and with the sum over excited singlets in equation S1 running over the 20 lowest states. Computations have been done for the molecular crystal structures at the ωB97X-D/6-31+G(d) level.

state	**CBZ-S**	**CBZ-SO**	**CBZ-SO$_2$**
T_1	$4.7\cdot10^{-9}$	$6.2\cdot10^{-9}$	$2.5\cdot10^{-8}$
T_2	$2.6\cdot10^{-9}$	$8.1\cdot10^{-9}$	$1.1\cdot10^{-8}$

Optimized geometries

CBZ-S: S_0 state

S	24.929617	1.689602	4.065272
C	24.794085	2.814206	2.686971
C	23.907706	3.895676	2.703404
H	23.311695	4.095619	3.588990
C	23.806723	4.732051	1.599733
C	24.556661	4.470791	0.450104
C	25.423712	3.380125	0.422228
C	25.557303	2.567225	1.544729
H	26.253992	1.734225	1.529416
C	25.065154	2.814206	5.443572
H	23.141118	5.589937	1.619348
N	24.436517	5.313492	-0.679729
H	25.998464	3.175939	-0.476225
C	24.301941	2.567225	6.585818
C	25.951531	3.895677	5.427136
C	23.258015	5.581133	-1.371620
C	25.473592	6.020986	-1.282257
H	23.605253	1.734223	6.601133
C	24.435536	3.380124	7.708319
H	26.547539	4.095621	4.541547
C	26.052518	4.732052	6.530806
C	21.974142	5.079241	-1.153623
C	23.538372	6.469123	-2.430806
C	24.958303	6.751507	-2.373217
C	26.820668	6.088544	-0.923667
H	23.860788	3.175938	8.606773
C	25.302585	4.470791	7.680438
H	26.718122	5.589939	6.511189
C	20.965764	5.500074	-2.012170
H	21.769294	4.380541	-0.348272
C	22.508111	6.877282	-3.280466
C	25.812769	7.554248	-3.131686
H	27.207238	5.537422	-0.071898
C	27.651057	6.894227	-1.693373
N	25.422733	5.313493	8.810271
C	21.224389	6.392938	-3.064263
H	19.956537	5.125819	-1.865850
H	22.709668	7.561218	-4.100480
H	25.429387	8.122307	-3.974984
C	27.157466	7.619020	-2.789663
H	28.704406	6.965093	-1.437512
C	24.385659	6.020985	9.412804
C	26.601238	5.581136	9.502157
H	20.412798	6.702129	-3.716025
H	27.834077	8.238425	-3.370686
C	23.038582	6.088540	9.054220
C	24.900952	6.751507	10.503761
C	27.885111	5.079246	9.284153
C	26.320884	6.469125	10.561343
H	22.652009	5.537417	8.202453
C	22.208195	6.894221	9.823930
C	24.046488	7.554246	11.262235
H	28.089956	4.380546	8.478802
C	28.893492	5.500080	10.142696
C	27.351149	6.877286	11.410999
H	21.154845	6.965086	9.568074

C	22.701790	7.619015	10.920218
H	24.429873	8.122306	12.105530
H	29.902719	5.125827	9.996372
C	28.634870	6.392944	11.194790
H	27.149594	7.561222	12.231013
H	22.025180	8.238420	11.501244
H	29.446464	6.702136	11.846549

CBZ-SO: S_0 state

S	26.082388	1.181616	3.991448
C	25.720475	2.382716	2.683268
C	24.402824	2.742458	2.420725
H	23.583931	2.297981	2.981302
C	24.136994	3.691988	1.439182
H	23.118258	4.008510	1.237036
C	25.188142	4.245220	0.705576
C	26.504475	3.845127	0.951801
C	26.773828	2.915738	1.948950
H	27.788015	2.581566	2.150361
O	27.455873	0.632756	3.713001
C	26.287885	2.406876	5.315942
N	24.920461	5.209314	-0.291868
H	27.308436	4.262281	0.352934
C	25.174737	2.998337	5.906214
C	27.575482	2.715839	5.734017
C	24.067884	5.043049	-1.381667
C	25.450161	6.497959	-0.336212
H	24.169114	2.730888	5.591024
C	25.356152	3.934399	6.916999
H	28.420424	2.218601	5.265643
C	27.755409	3.665778	6.734957
C	23.340464	3.917203	-1.769822
C	24.050709	6.234300	-2.135942
C	24.934953	7.166388	-1.465741
C	26.318930	7.126610	0.556798
H	24.502364	4.397091	7.402540
C	26.648126	4.277155	7.324846
H	28.754042	3.943806	7.058337
C	22.574908	4.014113	-2.925652
H	23.375733	2.994587	-1.198693
C	23.274153	6.307147	-3.294039
C	25.312230	8.486808	-1.718322
H	26.697152	6.614595	1.436235
C	26.679622	8.440833	0.284419
N	26.830177	5.242815	8.340375
C	22.535485	5.196377	-3.681194
H	21.998528	3.152940	-3.251417
H	23.252017	7.218785	-3.884962
H	24.923442	9.013667	-2.585520
C	26.187169	9.116995	-0.842761
H	27.355907	8.954230	0.961905
C	26.355912	6.552881	8.308372
C	27.520524	5.050697	9.535802
H	21.926331	5.238142	-4.579072
H	26.490846	10.143103	-1.026855
C	25.642886	7.209685	7.304392
C	26.743377	7.209738	9.494432
C	28.140916	3.899143	10.022108

C	27.488219	6.247369	10.280852
H	25.366030	6.706470	6.383091
C	25.306255	8.540635	7.520718
C	26.393227	8.547350	9.688907
H	28.145914	2.974364	9.453464
C	28.748211	3.974995	11.269834
C	28.105184	6.298774	11.532418
H	24.750981	9.076168	6.755978
C	25.671744	9.205902	8.701416
H	26.685059	9.065614	10.598304
H	29.239758	3.093939	11.672357
C	28.736533	5.161615	12.019638
H	28.087862	7.214602	12.117017
H	25.391498	10.245883	8.838983
H	29.221800	5.186407	12.990671

CBZ-SO_2: S_0 state

C	17.899985	12.089477	-0.413436
H	17.771094	11.098540	0.011200
H	19.978695	12.205189	0.146628
C	16.830384	12.720056	-1.036022
C	19.151543	12.719244	-0.334412
H	15.864332	12.227881	-1.107714
C	17.015498	13.994836	-1.574271
C	19.359853	13.987857	-0.862671
C	16.150748	14.909593	-2.292222
C	18.274963	14.619576	-1.471860
H	20.335364	14.460802	-0.807219
C	14.810070	14.860785	-2.678868
C	16.923861	16.048191	-2.596503
N	18.213412	15.866741	-2.094706
H	14.203160	13.988921	-2.450518
C	14.263713	15.944771	-3.353778
C	16.379920	17.149009	-3.259446
C	19.280367	16.782027	-2.208754
H	13.222844	15.921338	-3.661893
C	15.043592	17.077231	-3.634570
H	16.970097	18.035730	-3.468619
C	19.658511	17.258785	-3.466673
C	19.954568	17.211681	-1.062215
H	14.594770	17.919828	-4.152560
C	20.709739	18.160302	-3.580184
H	19.131646	16.913531	-4.350697
H	19.631202	16.858550	-0.088071
C	21.017328	18.099179	-1.173357
H	21.004694	18.549708	-4.549412
C	21.385287	18.565206	-2.432646
H	21.538203	18.453311	-0.289368
S	22.778332	19.675083	-2.582790
O	22.639736	20.386257	-3.851304
C	24.171376	18.565206	-2.732934
O	22.916927	20.386257	-1.314277
C	24.539335	18.099180	-3.992224
C	24.846924	18.160302	-1.585397
H	24.018460	18.453311	-4.876213
C	25.602095	17.211681	-4.103366
H	24.551969	18.549708	-0.616169
C	25.898152	17.258785	-1.698908

H	25.925461	16.858550	-5.077509
C	26.276296	16.782027	-2.956826
H	26.425016	16.913530	-0.814884
N	27.343251	15.866741	-3.070875
C	27.281701	14.619576	-3.693721
C	28.632803	16.048191	-2.569078
C	26.196810	13.987857	-4.302910
C	28.541165	13.994836	-3.591309
C	29.405915	14.909593	-2.873358
C	29.176743	17.149009	-1.906134
C	26.405120	12.719244	-4.831169
H	25.221299	14.460802	-4.358362
C	28.726279	12.720056	-4.129559
C	30.746593	14.860785	-2.486713
H	28.586566	18.035730	-1.696962
C	30.513071	17.077231	-1.531011
H	25.577968	12.205189	-5.312209
C	27.656678	12.089478	-4.752145
H	29.692331	12.227881	-4.057867
H	31.353503	13.988921	-2.715063
C	31.292950	15.944771	-1.811802
H	30.961893	17.919828	-1.013020
H	27.785569	11.098540	-5.176781
H	32.333819	15.921338	-1.503687

References

1. Cheng, Z.; Sun, P.; Tang, A.; Jin, W.; Liu, C. *Org. Lett.* **2019**, *21*, 8925–8929.
2. Dolomanov, O. V.; Bourhis, L. J.; Gildea, R. J.; Howard, J. A. K.; Puschmann, H.; *J. Appl. Cryst.* **2009**, *42*, 339–341.
3. Sheldrick, G. M. *Acta Cryst.* **2015**, *A71*, 3–8.
4 . L. Xu, K. Zhou, H. Ma, A. Lv, D. Pei, G. Li, Y. Zhang, Z. An, A. Li and G. He, *ACS Appl Mater Interfaces*, 2020, **12**, 18385–18394.
5. S. Xu, T. Liu, Y. Mu, Y.-F. Wang, Z. Chi, C.-C. Lo, S. Liu, Y. Zhang, A. Lien and J. Xu, *Angew. Chem. Int. Ed.*, 2014, **54**, 874–878.
6. Chai, J.-D.; Head-Gordon, M. *Phys Chem Chem Phys* **2008**, *10*, 6615–6620.
7. Liu, F.; Livshits, E.; Lochan, R. C.; Luenser, A.; Manohar, P.; Manzer, S. F.; Mao, S.-P.; Mardirossian, N.; Marenich, A. V.; Maurer, S. A.; Mayhall, N. J.; Neuscamman, E.; Oana, C. M.; Olivares-Amaya, R.; O'Neill, D. P.; Parkhill, J. A.; Perrine, T. M.; Peverati, R.; Prociuk, A.; Rehn, D. R.; Rosta, E.; Russ, N. J.; Sharada, S. M.; Sharma, S.; Small, D. W.; Sodt, A.; Stein, T.; Stueck, D.; Su, Y.-C.; Thom, A. J. W.; Tsuchimochi, T.; Vanovschi, V.; Vogt, L.; Vydrov, O.; Wang, T.; Watson, M. A.; Wenzel, J.; White, A.; Williams, C. F.; Yang, J.; Yeganeh, S.; Yost, S. R.; You, Z.-Q.; Zhang, I. Y.; Zhang, X.; Zhao, Y.; Brooks, B. R.; Chan, G. K. L.; Chipman, D. M.; Cramer, C. J.; Goddard, W. A.;

Gordon, M. S.; Hehre, W. J.; Klamt, A.; Schaefer, H. F.; Schmidt, M. W.; Sherrill, C. D.; Truhlar, D. G.; Warshel, A.; Xu, X.; Aspuru-Guzik, A.; Baer, R.; Bell, A. T.; Besley, N. A.; Chai, J.-D.; Dreuw, A.; Dunietz, B. D.; Furlani, T. R.; Gwaltney, S. R.; Hsu, C.-P.; Jung, Y.; Kong, J.; Lambrecht, D. S.; Liang, W.; Ochsenfeld, C.; Rassolov, V. A.; Slipchenko, L. V.; Subotnik, J. E.; Van Voorhis, T.; Herbert, J. M.; Krylov, A. I.; Gill, P. M. W.; Head-Gordon, M.; Shao, Y.; Gan, Z.; Epifanovsky, E.; Gilbert, A. T. B.; Wormit, M.; Kussmann, J.; Lange, A. W.; Behn, A.; Deng, J.; Feng, X.; Ghosh, D.; Goldey, M.; Horn, P. R.; Jacobson, L. D.; Kaliman, I.; Khaliullin, R. Z.; Kus, T.; Landau, A.; Liu, J.; Proynov, E. I.; Rhee, Y. M.; Richard, R. M.; Rohrdanz, M. A.; Steele, R. P.; Sundstrom, E. J.; Woodcock, H. L.; Zimmerman, P. M.; Zuev, D.; Ben Albrecht; Alguire, E.; Austin, B.; Beran, G. J. O.; Bernard, Y. A.; Berquist, E.; Brandhorst, K.; Bravaya, K. B.; Brown, S. T.; Casanova, D.; Chang, C.-M.; Chen, Y.; Chien, S. H.; Closser, K. D.; Crittenden, D. L.; Diedenhofen, M.; Distasio, R. A.; Do, H.; Dutoi, A. D.; Edgar, R. G.; Fatehi, S.; Fusti-Molnar, L.; Ghysels, A.; Golubeva-Zadorozhnaya, A.; Gomes, J.; Hanson-Heine, M. W. D.; Harbach, P. H. P.; Hauser, A. W.; Hohenstein, E. G.; Holden, Z. C.; Jagau, T.-C.; Ji, H.; Kaduk, B.; Khistyaev, K.; Kim, J.; Kim, J.; King, R. A.; Klunzinger, P.; Kosenkov, D.; Kowalczyk, T.; Krauter, C. M.; Lao, K. U.; Laurent, A. D.; Lawler, K. V.; Levchenko, S. V.; Lin, C. Y. *Molecular Physics* **2015**, *113*, 184–215.
